# *Drosophila* HisT is a specific histamine transporter that contributes to histamine recycling in glia

**DOI:** 10.1126/sciadv.abq1780

**Published:** 2022-10-26

**Authors:** Jun Xie, Yongchao Han, Yufeng Liang, Lei Peng, Tao Wang

**Affiliations:** ^1^ National Institute of Biological Sciences, Beijing 102206, China.; ^2^ School of Life Sciences, Beijing Normal University, Beijing 100875, China.; ^3^ College of Biological Sciences, China Agricultural University, Beijing 100083, China.; ^4^ Tsinghua Institute of Multidisciplinary Biomedical Research, Tsinghua University, Beijing 100084, China.

## Abstract

Histamine is an important monoamine neurotransmitter that regulates multiple physiological activities in both vertebrates and invertebrates. Clearance and recycling of histamine are critical for sustaining histaminergic transmission. However, unlike other monoamine neurotransmitters, a histamine-specific transporter capable of clearing histamine from the synaptic cleft has not been identified. Here, through an in vitro histamine uptake screening, we identified an epithelial glia–expressing transporter, HisT (Histamine Transporter), that specifically transports histamine into cells. HisT misexpression in both pre- and postsynaptic neurons revealed a critical in vivo role for HisT in histamine transport and synaptic transmission. Last, we generated null *hist* alleles and demonstrated key physiological roles of HisT in maintaining histamine pools and sustaining visual transmission when the de novo synthesis of histamine synthesis was reduced. Our work identifies the first transporter that specifically recycles histamine and further indicates that the histamine clearance pathway may involve both the uptake-1 and uptake-2 transport systems.

## Teaser

HisT is a sodium-dependent histamine transporter with substrate specificity in *Drosophila* glial cells.

## INTRODUCTION

Neurotransmitters are chemical messengers that enable the transmission of electrical signals between neurons. After release into the synaptic cleft, neurotransmitters are rapidly removed to avoid overstimulation of neurotransmitter receptors located on postsynaptic neurons. Impaired neurotransmitter removal results in severe brain disorders, including epilepsy, depression, addiction, and neurodegenerative diseases (*1*–*3*). The critical mechanism by which most neurotransmitters, in particular monoamine neurotransmitters (e.g., dopamine, norepinephrine, serotonin, and histamine), are removed is through reuptake transporters within the plasma membrane of presynaptic neurons and astrocytes (*4*, *5*).

Two transport systems are responsible for the clearance of extracellular monoamine neurotransmitters within the central nervous system, namely, the uptake-1 and uptake-2 systems. Uptake-1 transporters have high affinity and low capacity, and are ion dependent, whereas uptake-2 transporters have low affinity and high capacity, and are ion independent (*6*, *7*). Uptake-1 transporters include DAT (dopamine transporter), SERT (serotonin transporter), and NET (norepinephrine transporter), which are responsible for the specific reuptake of extraneuronal dopamine, serotonin, and norepinephrine, respectively, in a sodium/chloride-dependent manner (*8*). Drugs that inhibit these uptake-1 transporters (e.g., serotonin-selective reuptake inhibitors) are used to treat patients suffering from neurological disorders such as depression, anxiety, and addiction (*9*). By contrast, uptake-1 transporters specific for histamine have not been identified, suggesting that the uptake-2 system may be involved in clearing histamine (*10*, *11*). These transporters include OCT2 (organic cation transporter 2), OCT3, and PMAT (plasma membrane monoamine transporter) (*12*–*16*). However, the low specificity of these transporters makes pharmacological regulation of histamine clearance extremely difficult when trying to treat neurological disorders such as neuropathic pain, Tourette’s syndrome, and myoclonic dystonia (*17*, *18*). Furthermore, genetic deletion of OCT2, OCT3, or PMAT in mice failed to indicate direct roles for these type 2 transporters in histamine transmission in vivo (*15*, *19*, *20*).

Histamine is an important neurotransmitter in the brain, as histaminergic neurons project axons from the tuberomammillary nucleus of the posterior hypothalamus to the entire brain to modulate diverse physiological functions such as sleep-wake cycles, learning and memory, synaptic plasticity, and feeding behaviors (*21*, *22*). Unlike other monoamines, whose reuptake and recycling occur in presynaptic neurons, it has been proposed that histamine is taken up and inactivated primarily in adjacent astrocytes (*11*, *23*, *24*). In *Drosophila*, histamine is a major neurotransmitter in primary photoreceptor neurons, and in this context, it is clear that histamine is cleared primarily by laminar epithelial glia. Within these glial cells, histamine is converted to its inactive form, carcinine, by the *N*-β-alanyl-dopamine synthase Ebony (*25*–*30*). Carcinine is then transported back into photoreceptors via the uptake-2 transporter, CarT, and hydrolyzed back to histamine by the *N*-β-alanyl-dopamine hydrolase Tan. Last, histamine is loaded into synaptic vesicles by the vesicular histamine transporter, LOVIT (loss of visual transmission), thereby restoring the histamine pool (*25*, *31*–*34*). Although most details of this histamine recycling pathway have been established, the transporter responsible for the uptake of histamine into epithelial glia has not been identified.

Given that the enzyme responsible for inactivating histamine is only expressed by epithelial glia, we hypothesized that epithelial glia would also express a histamine-specific transporter that would be required for histamine clearance and recycling. Here, we report that the gene, *hist*, is enriched in epithelial glial and encodes a plasma membrane transporter capable of transporting histamine into cells. The HisT transporter is Na^+^ dependent and specific to histamine, indicating that HisT is an uptake-1 transporter. Overexpression and genetic mutation of *hist* confirm in vivo that HisT is a histamine transporter and that it plays a key physiological role in maintaining histamine pools and sustaining visual transmission.

## RESULTS

### CG1358 transports histamine in vitro

**Fig. 1.**
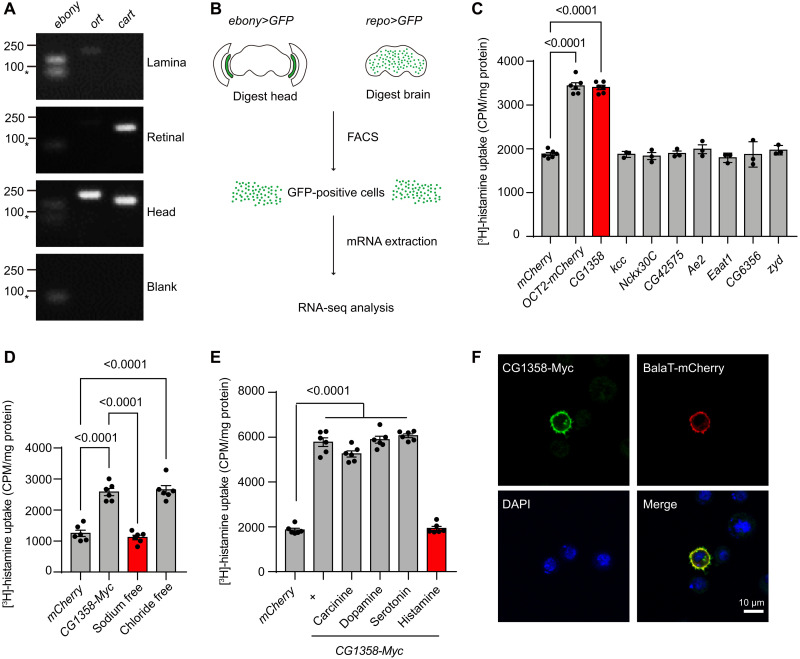
CG1358 is an epithelial glia–enriched histamine transporter. (**A**) Strategy to screen the lamina-expressed candidates from head-specific transporter genes by RT-PCR. *Ebony*, epithelial glia; *ort*, LMC neuron; *cart*, photoreceptor. (**B**) Procedure to identify epithelial glia–enriched transporters. FACS was applied to isolate GFP-expressing epithelial cells from dissected *ebony>GFP* heads. As a control, the central brains of *repo>GFP* flies were dissected and trypsin-digested. GFP-expressing central brain glial cells were then isolated via FACS. mRNA of collected cells was extracted, and RNA-seq analysis was performed. (**C**) Screening the candidate transporters identified as both lamina-expressed and epithelial glia–enriched genes for histamine transporter in vitro. One of eight overlapping transporters was transiently expressed in HEK293T cells, and [^3^H]-histamine was added to the ECF buffer (final concentration, 2.5 μM). Human OCT2 and mCherry were used as positive and negative controls, respectively. Results are mean values ± SEM of six experiments for OCT2, mCherry, and CG1358 and three experiments for other candidates. CPM, counts per minute. (**D**) Histamine transport by CG1358 depends on Na^+^ but not Cl^−^. CG1358 was transiently expressed in HEK293T cells, followed by incubation with ECF, Na^+^-free ECF, or Cl^−^-free ECF buffer containing [^3^H]-histamine. mCherry was used as a control. Results are mean values ± SEM for six experiments. (**E**) CG1358 is a specific histamine transporter. HEK293T cells transiently expressing CG1358 were subjected to competition assays using [^3^H]-histamine in combination with higher concentrations of different monoamines, namely, histamine, carcinine, dopamine, and serotonin (0.1 mM for serotonin and dopamine and 0.5 mM for histamine and carcinine versus 2.5 μM [^3^H]-histamine). mCherry was used as a control. Results are mean values ± SEM for six experiments. (**F**) S2 cells were transiently cotransfected with Myc-tagged CG1358 and mCherry-tagged BalaT and were labeled with Myc antibody (green), mCherry antibody (red), and 4′,6-diamidino-2-phenylindole (DAPI; blue). Scale bar, 10 μm.

To identify a histamine transporter in the fly visual system, we speculated that a transporter responsible for histamine uptake would likely be expressed in epithelial glial cells within the lamina that also express ebony. Thus, we examined previous RNA sequencing (RNA-seq) data that compared mRNAs isolated from the head with mRNAs isolated from the body (*35*). Among ~600 putative transmembrane transporters in *Drosophila*, there are 62 putative transporters enriched in fly head (*36*). To test whether those transporters are expressed in lamina, we dissected fly head, retinal, and lamina, respectively, and detected transporter expression by reverse transcription polymerase chain reaction (RT-PCR) (Fig. 1A). To verify the purity of the dissected tissues, we examined the expression of *ebony* and *ort*, which are laminar expressing, and *cart*, which is retinal expressing. As expected, both *ebony* and *ort* are expressed in lamina and head, without retinal expressing, while *cart* is specifically expressed in retinal and head (Fig. 1A). We identified 24 lamina-expressed transporters that represented potential histamine transporters (Table 1).

**Table 1.** Histamine transporter candidates. ATP, adenosine 5′-triphosphate; N/A, not available.

**Gene**	***#CG***	**Description**	**Histamine uptake**
Overlapped transporters			
*kcc*	*CG5594*	slc12 family of cation-coupled chloride transporters	No
*Nckx30C*	*CG18660*	slc24 family of potassium-dependent sodium/calcium exchangers	No
*CG42575*	*CG42575*	slc20 family of sodium/phosphate cotransporters	No
*Ae2*	*CG8177*	slc4 family of bicarbonate transporters	No
*Eaat1*	*CG3747*	slc1 family of glutamate and neutral amino acid transporters	No
*CG1358*	*CG1358*	slc49 family of flvcr-related heme transporters	Yes
*CG6356*	*CG6356*	slc22 family of organic ion transporters	No
*zyd*	*CG2893*	slc24 family of potassium-dependent sodium/calcium exchangers	No
Lamina-enriched transporters			
*Ent3*	*CG11010*	Monoamine transmembrane transporter/SLC29A4	No
*mdr65*	*CG10181*	ATP-binding cassette (ABC) transporters	No
*CG1090*	*CG1090*	Potassium-dependent sodium/calcium exchangers	No
*hoe1*	*CG12787*	Membrane transporter	No
*CG13178*	*CG13178*	Organic solute carrier	No
*CG13795*	*CG13795*	Sodium/chloride-dependent neurotransmitter transporters/SLC6	No
*CG15890*	*CG15890*	Folate transporter–like family/SLC46	No
*atet*	*CG2969*	ATP-binding cassette (ABC) transporters	No
*CG31663*	*CG31663*	Major facilitator superfamily	No
*VAChT*	*CG32848*	Vesicular neurotransmitter transporter/SLC18	No
*balat*	*CG3790*	Organic ion transporter/SLC22	No
*cngl*	*CG43395*	Intracellular cyclic nucleotide–activated cation channel	No
*ncc69*	*CG4357*	Cation-coupled chloride transporters/SLC12	No
*lovit*	*CG45782*	Vesicle histamine transporter, sucrose transporter/SLC45	N/A
*CG5888*	*CG5888*	Potassium channel	No
*CG7342*	*CG7342*	Organic ion transporters/SLC22	No
Epithelial glia–enriched transporters			
*CG3036*	*CG3036*	slc17 family of organic anion transporters	No
*CG18549*	*CG18549*	mfs transporter superfamily	No
*Picot*	*CG8098*	slc17 family of organic anion transporters	No
*CG6812*	*CG6812*	slc56 family of sideroflexins	No
*CG11857*	*CG11857*	mfs transporter	No
*path*	*CG3424*	slc36 family of proton-coupled amino acid transporters	N/A
*VGlut*	*CG9887*	slc17 family of organic anion transporters	N/A
*CG8195*	*CG8195*	slc35 family of nucleotide sugar transporters	No
*Ndae1*	*CG42253*	slc4 family of bicarbonate transporters	No
*Nhe3*	*CG11328*	slc9 family of sodium/proton exchangers	No
*hrm*	*CG11665*	slc16 family of monocarboxylate transporters	No
*MFS17*	*CG40263*	slc17 family of organic anion transporters	N/A
*CG43066*	*CG43066*	slc6 family of sodium/chloride-dependent neurotransmitter transporters	No
*CG10444*	*CG10444*	slc5 family of sodium/glucose cotransporter transporters	No
*CG6126*	*CG6126*	slc22 family of organic ion transporters	N/A
*blot*	*CG3897*	slc6 family of sodium/chloride-dependent neurotransmitter transporters	No
*Eaat2*	*CG3159*	slc1 family of glutamate and neutral amino acid transporters	N/A
*CG6006*	*CG6006*	slc22 family of organic ion transporters	N/A
*CG5281*	*CG5281*	slc35 family of nucleotide sugar transporters	No
*CG7708*	*CG7708*	slc5 family of sodium/glucose cotransporter transporters	No
*CG13384*	*CG13384*	slc36 family of proton-coupled amino acid transporters	No
*CG10804*	*CG10804*	slc6 family of sodium/chloride-dependent neurotransmitter transporters	No
*mnd*	*CG3297*	L-type amino acid transporters	N/A
*kar*	*CG12286*	slc16 family of monocarboxylate transporters	No

To further identify transporters that are enriched in epithelial glia, we generated *ebony^Gal4^* knock-in flies, and by combination of *UAS-GFP* (*ebony>GFP*), we expressed GFP (green fluorescent protein) specifically in epithelial glia (fig. S2, A and B). After dissection and trypsin digestion of heads of *ebony>GFP* flies, GFP-positive cells were isolated by FACS (fluorescence-activated cell sorting), and RNA-seq was performed to verify epithelial glia–enriched genes. We used central brain glial cells isolated via FACS from *repo>GFP* (*repo-Gal4/UAS-GFP*) central brains (without lamina) as a control (Fig. 1B). Using this method, we identified 32 epithelial glia–enriched transporters that represented potential histamine transporters (Table 1). Further comparing the 24 lamina-expressed transporters and 32 epithelial glia–enriched transporters, we found eight genes encoding putative transporters identified in both methods, including *CG1358*, *Kcc*, *Nckx30C*, *CG42575*, *Ae2*, *Eaat1*, *CG6356*, and *Zyd*, which are top candidate histamine transporters (fig. S1A).

To determine whether these candidate transporters could transport histamine, we expressed 40 candidate transporters in human embryonic kidney (HEK) 293T cells and conducted histamine uptake assays. As a positive control, the human OCT (OCT2), which mediates the low-affinity transport of some monoamine neurotransmitters including histamine, exhibited high levels of histamine transport activity when expressed in HEK293T cells (*14*). Among all candidate transporters, CG1358, which is identified as an epithelial glia–enriched gene by both methods above, is the only transporter exhibiting histamine uptake activity in vitro (Fig. 1C, Table 1, and fig. S1, B and C). To further characterize CG1358, we next examine the specificity and Na^+^ and/or Cl^−^ dependency of the CG1358 histamine transporter. As results, we found that the histamine uptake activity by CG1358 depends on Na^+^ but not Cl^−^, indicating that the histamine uptake activity by CG1358 is an active transport (Fig. 1D). The known histamine transporter including OCT2 belongs to the uptake-2 system transporter with ion independency, as OCT2 is able to transport histamine regardless of the presence of Na^+^ and Cl^−^ (fig. S1D) (*31*). To examine whether CG1358 is specific to histamine, we performed competition assays using [^3^H]-histamine in combination with different monoamines at high concentration. As a consequence, histamine could efficiently block [^3^H]-histamine uptake, whereas other monoamines including dopamine, serotonin, and carcinine, the inactive form of histamine, could not affect the histamine uptake activity of CG1358, suggesting that CG1358 is a high-affinity histamine transporter (Fig. 1E). In contrast, the uptake-2 transporter OCT2 showed little substrate selectivity toward monoamines, as OCT2-mediated [^3^H]-histamine uptake was completely blocked by dopamine, serotonin, and carcinine, but not by the amino acid β-alanine, the other key intermediate of histamine recycle (fig. S1E). Moreover, when we expressed Myc-tagged CG1358 in S2 cells, the Myc signal localized to the plasma membrane (Fig. 1F). Together, our results suggest that CG1358 encodes an epithelial glia–enriched plasma membrane histamine transporter; we therefore named this gene *hist* (*histamine transporter*).

### HisT localizes specifically to epithelial glial cells

Upon release to the synaptic cleft, histamine is quickly transported to the epithelial glial cells, where histamine is inactivated by Ebony before recycling to photoreceptor cells. On this purpose, histamine transporter should be coupled with Ebony and expressed exclusively in Ebony-expressing epithelial glial cells. To visualize the epithelial glial cell, we knocked the red fluorescent protein mCherry into the *ebony* locus via CRISPR-Cas9 genome editing, resulting in mCherry expression driven by the native *ebony* promoter (*ebony^mCherry^*) (fig. S2, A and B). Electroretinogram (ERG) recordings measure the summed responses of compound eyes in response to light, including a sustained depolarizing response from the photoreceptors, and on- and off-transients that depend on synaptic transmission to the laminal LMC (large monopolar cell) neurons (*31*). The *ebony^mCherry^* flies completely lost on- and off-transients in ERG response and Ebony immunosignals (fig. S2, C to E) (*37*). Moreover, the *ebony^mCherry^* flies also exhibit dark body color phenotypes (fig. S2F). These results demonstrated that *ebony^mCherry^* was an *ebony* loss-of-function allele, and we used *ebony^mCherry^* as *ebony* mutant since it has the same genetic background as other mutants generated.

**Fig. 2.**
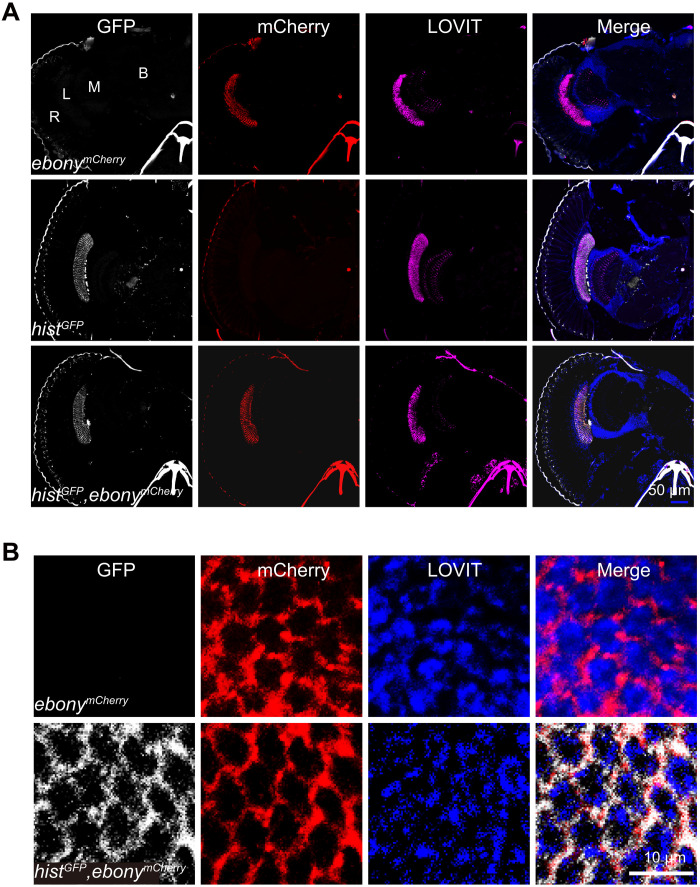
HisT localizes to epithelial glial cells. (**A** and **B**) Cryosections were prepared from *hist^GFP^;ebony^mCherry^* double–knock-in flies, in which the expression of GFP and mCherry was driven by the native promoter of *hist* and *ebony*, respectively. The samples were labeled for GFP (gray), mCherry (red), and the synaptic vesicle marker LOVIT [magenta in (A) and blue in (B)]. Scale bars, 50 μm (A) and 10 μm (B). L, lamina; M, medulla; R, retina; B, brain.

Because we failed to generate anti-HisT antibodies effective for both immunostaining and Western blots, to determine the expression pattern of *hist*, using CRISPR-Cas9 genome editing, we created a GFP knock-in allele, in which GFP expression is driven by the native *hist* promoter (*hist^GFP^*) (fig. S3, A and B). First, from RT-PCR, we determined that HisT was specifically expressed in the adult eye, with no expression detected in the brain or body (fig. S3C). Consistent with these mRNA expression results, the GFP signal in cryosectioned *hist^GFP^* heads was detected exclusively in the lamina, not in the retina, medulla, or brain. Furthermore, in *hist^GFP^;ebony^mCherry^* double–knock-in flies, we found that the *hist-*GFP signal completely colocalized with the *Ebony-*mCherry signal in epithelial glial cells (Fig. 2, A and B). The finding that HisT was coexpressed with Ebony in epithelial glial cells is consistent with our hypothesis that HisT functions as a histamine-specific transporter in vivo.

### Dysregulating histamine transport disrupts synaptic transmission

HisT belongs to the solute carrier 49 (SLC49) family and shares 52.37 and 51.02% identity with the feline leukemia virus subgroup C receptor–related protein 2 (FLVCR2) and FLVCR1, respectively (fig. S4A). FLVCR1 mutations have been documented in patients with a rare neurodegenerative disorder, posterior column ataxia with retinitis pigmentosa, whereas FLVCR2 mutations are associated with Fowler syndrome, a rare proliferative vascular disorder of the brain (*38*). We found that FLVCR1 and FLVCR2 did not exhibit histamine uptake activity in vitro (fig. S4B). Both FLVCR1 and FLVCR localized to the plasma membrane, confirming the proper localization of these two transporters (fig. S4C). However, most residues that mutated in FLVCR1 and FLVCR2 in the context of disease are conserved in HisT, suggesting that these sites may play critical roles in substrate transport (fig. S4, A and B). We therefore asked whether these conserved sites are critical for HisT-mediated histamine transport. Mutating these conserved sites in HisT impaired its ability to transport histamine in vitro (fig. S4D). To exclude the possibility that these mutated HisT proteins were mislocalized, we assessed the localization of HisT^L109P^ and HisT^P252R^ in S2 cells and confirmed appropriate membrane localization (fig. S4E).

**Fig. 3.**
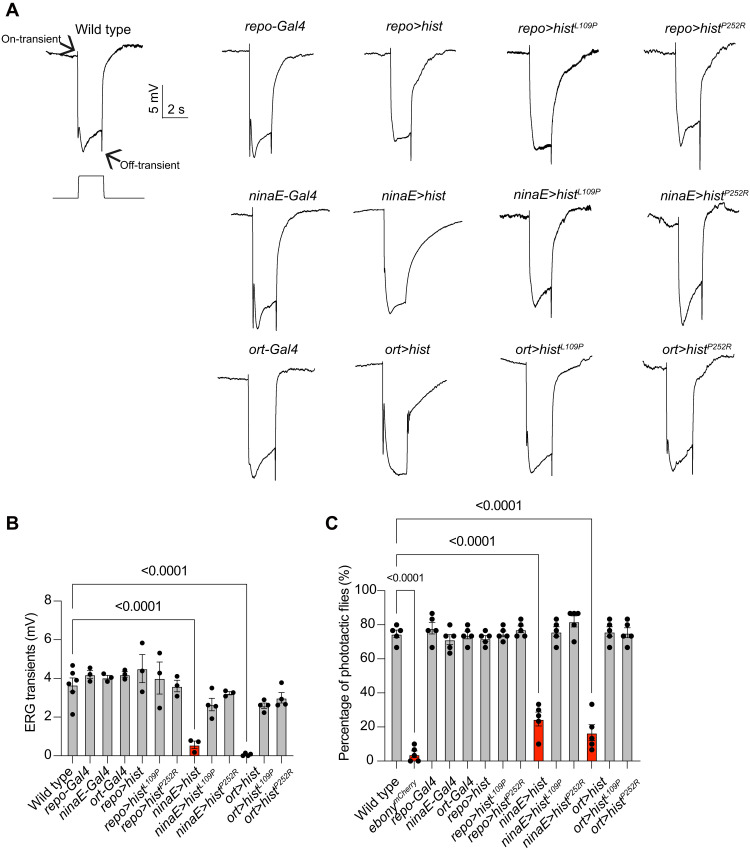
Misexpression of HisT disrupts synaptic transmission. (**A**) ERG recordings from wild-type (*w^1118^*), *repo-Gal4*, *repo>hist* (*repo-Gal4*/*UAS-hist*), *repo>hist^L109P^* (*repo-Gal4*/*UAS-hist^L109P^*), *repo>hist^P252R^* (*repo-Gal4*/*UAS-hist^P252R^*), *ninaE-Gal4*, *ninaE>hist* (*ninaE-Gal4*/*UAS-hist*), *ninaE>hist^L109P^* (*ninaE-Gal4*/*UAS-hist^L109P^*), *ninaE>hist^P252R^* (*ninaE-Gal4*/*UAS-hist^P252R^*), *ort-Gal4*, *ort>hist* (*ort-Gal4*/*UAS-hist*), *ort>hist^L109P^* (*ort-Gal4*/*UAS-hist^L109P^*), and *ort>hist^P252R^* (*ort-Gal4*/*UAS-hist^P252R^*) flies. Young flies (<3 days after eclosion) were dark-adapted for 1 min and subsequently exposed to a 2-s pulse of orange light. The on- and off-transients are indicated by arrows. (**B**) Quantitative analysis of the amplitudes of ERG off-transients shown in (A). (**C**) Phototactic behaviors of flies corresponding to those in (A), and *ebony* mutant (*ebony^mCherry^*) was used as a positive control. At least 20 flies at 3 days old were used, and five repeats were quantified for each group. Significant differences between mutant and wild-type flies were determined using unpaired *t* tests.

To determine whether HisT functions as a histamine transporter in vivo, we first misexpressed HisT in photoreceptor cells using a combination of *UAS-hisT* and *ninaE*-*Gal4* (*neither inactivation nor afterpotential E*, which encodes the major rhodopsin). We then asked whether mislocalization of HisT could disrupt visual transmission, as measured by ERG recordings. We found that on- and off-transients were reduced in *ninaE>hisT* flies. We next misexpressed HisT in postsynaptic LMC neurons. We first knocked in *Gal4* sequence into the *ort* locus (*ora transientless*, which encodes LMC neuron–specific histamine-gated chloride channel) by CRISPR-Cas9 genome editing, in which Gal4 expression is limited to LMC neurons in both lamina and medulla (fig. S5, A to C). When LMC neurons were induced to express HisT by *ort-Gal4*, on- and off-transients were reduced, whereas flies expressing HisT in glial cells via *repo-Gal4* (*reversed polarity*) exhibited normal on- and off-transients (Fig. 3, A and B, and fig. S7A). Furthermore, expressing transport-dead versions of HisT, namely, HisT^L109P^ and HisT^P252R^, in photoreceptors and postsynaptic LMC neurons had no effect on ERG transients, suggesting that disruption of visual transmission by HisT misexpressing depends on its ability to transport histamine (Fig. 3, A and B). Consistent with these electrophysiology results, expressing HisT in photoreceptor cells and LMC neurons impaired phototactic behaviors. By contrast, flies expressing wild-type HisT in glial cells or mutant HisT^L109P^ or HisT^P252R^ in all three cell types exhibited normal phototaxis (Fig. 3C).

**Fig. 4.**
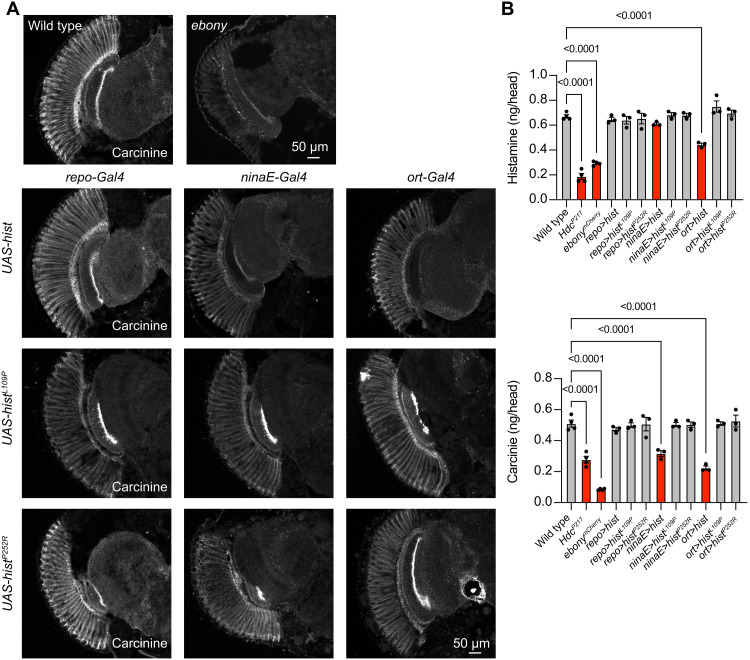
Misexpression of HisT disrupts histamine recycling. (**A**) Carcinine was immunolabeled in horizontal sections of heads from wild-type (*w^1118^*), *repo>hist*, *repo>hist^L109P^*, *repo>hist^P252R^*, *ninaE>hist*, *ninaE>hist^L109P^*, *ninaE>hist^P252R^*, *ort>hist*, *ort>hist^L109P^*, and *ort>hist^P252R^* flies, and *ebony* mutant was used as a positive control. Scale bars, 50 μm. (**B**) Levels of histamine and carcinine in the heads of wild-type, *repo>hist*, *repo>hist^L109P^*, *repo>hist^P252R^*, *ninaE>hist*, *ninaE>hist^L109P^*, *ninaE>hist^P252R^*, *ort>hist*, *ort>hist^L109P^*, and *ort>hist^P252R^* flies are shown. *Hdc* (*Hdc^P217^*) and *ebony* mutants were used as a positive control. Each sample contained 50 heads, and the mean values from three samples were calculated for each genotype. Error bars indicate SEMs, and significant differences between mutant and wild-type flies were determined using unpaired *t* tests.

To further characterize the effect of HisT misexpression on histamine signaling and metabolism, we examined in vivo levels of histamine and carcinine. The concentration of histamine was unaffected when HisT was misexpressed in photoreceptors, but carcinine levels were significantly reduced in these flies (Fig. 4, A and B). These results indicated that when HisT was expressed in photoreceptor cells, it rapidly cleared histamine into photoreceptors. As a consequence, levels of histamine were reduced in the synaptic cleft, resulting in less activation of downstream ORT channels. When HisT was misexpressed in LMC neurons, levels of both histamine and carcinine were reduced, supporting the hypothesis that transporting histamine into LMC cells impairs the normal histamine recycling process (Fig. 4, A and B). In both cases, uptake of histamine into epithelial glia was reduced, and thus, Ebony generated less carcinine. To confirm this reduction in carcinine, we stained head longitudinal sections with anticarcinine antibodies and found that carcinine levels were markedly reduced when HisT was expressed in photoreceptors or LMC cells but not in glial cells (Fig. 4, A and B). By contrast, expression of the transporter-dead HisT mutants, HisT^L109P^ and HisT^P252R^, in photoreceptor cells or LMC neurons did not affect the distribution or concentration of histamine or carcinine, further confirming that HisT transports histamine in vivo.

### Loss of *hist* does not impair histamine-mediated signaling under normal conditions

**Fig. 5.**
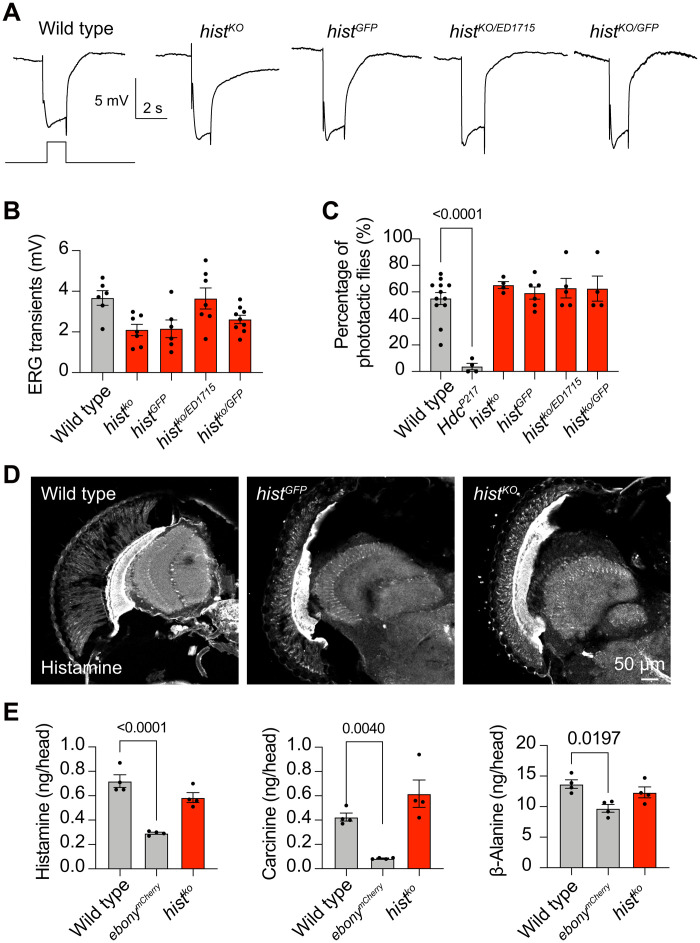
*hist* mutants display normal synaptic transmission. (**A**) ERG recordings from wild-type (*w^1118^*), *hist^ko^*, *hist^GFP^*, *hist^ko/ED1715^*, and *hist^ko/GFP^* flies. Young flies (<3 days after eclosion) were dark-adapted for 1 min and subsequently exposed to a 2-s pulse of orange light. (**B**) Quantitative analysis of the amplitudes of ERG off-transients shown in (A). (**C**) Phototactic behaviors of wild-type, *hist^ko^*, *hist^GFP^*, *hist^ko/ED1715^*, and *hist^ko/GFP^* flies. *Hdc* (*Hdc^P217^*) mutant was used as a positive control. At least 20 flies were used for each group, and five repeats were quantified for each group. Significant differences between mutant and wild-type flies were determined using unpaired *t* tests. (**D**) Histamine was immunolabeled in horizontal sections of heads from wild-type, *hist^ko^*, and *hist^GFP^* flies. Scale bar, 50 μm. (**E**) Levels of histamine, carcinine, and β-alanine in the heads of wild-type, *ebony^mCherry^*, and *hist^ko^* flies are shown. Each sample contained 50 heads, and the mean values from three samples were calculated for each genotype. Error bars indicate SEMs, and significant differences between mutant and wild-type flies were determined using unpaired *t* tests.

To study the physiological function of HisT in visual perception, we generated a null mutation of the *hist* gene by deleting a ~2800–base pair (bp) genomic fragment using the CRISPR-Cas9 system (fig. S5, D to G). PCR amplification of the *hist* locus from genomic DNA isolated from wild-type or *hist^ko^* flies revealed a truncated *hist* locus in mutant samples. In addition, for *hist^GFP^* knock-in flies, GFP sequences replace the first exon downstream of the start ATG. Therefore, both *hist^ko^* and *hist^GFP^* are considered *hist* null alleles. Unexpectedly, however, both *hist^GFP^* and *hist^ko^* mutants exhibited normal ERG responses, indicating normal synaptic transmission in the visual system of *hist* mutants (Fig. 5, A and B, and fig. S7B). We also assessed the ERG response of *hist^ko^* flies in response to high-frequency light stimuli and found that *hist^ko^* flies exhibited normal ERG transients (fig. S3D). To confirm these results, we performed ERG recording in *hist^ko^*/*hist^GFP^* transheterozygotes and again observed normal visual responses (Fig. 5, A and B, and fig. S7B). *hist* mutants also exhibited normal phototaxis, whereas *Hdc^P217^* flies, in which the de novo synthesis of histamine is disrupted, failed to exhibit phototactic behavior (Fig. 5C). To determine whether *hist* mutation affects histamine metabolism, we examined the distribution and concentration of histamine, carcinine, and β-alanine in *hist* null mutant flies. The distribution and concentration of these molecules did not change in *hist^GFP^* or *hist^ko^* mutant flies (Fig. 5, D and E). These data demonstrate that loss of *hist* alone does not affect histamine-mediated signaling, suggesting functional redundancy in the transport of histamine under normal conditions.

### HisT maintains visual transmission when the de novo synthesis of histamine is limited

**Fig. 6.**
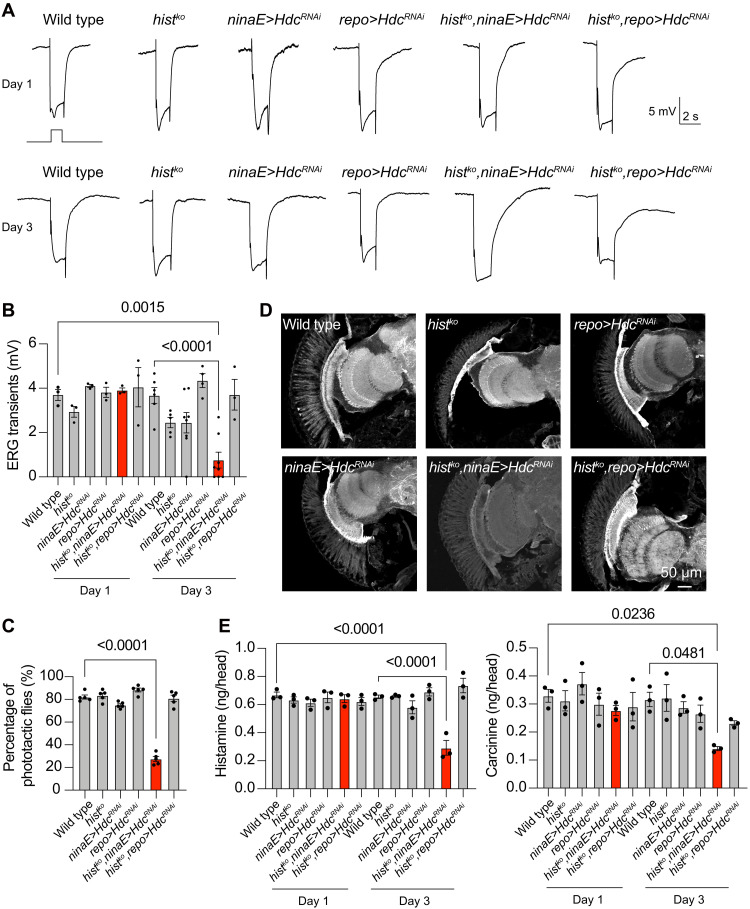
HisT maintains visual transmission when the de novo synthesis of histamine is disrupted. (**A**) ERG recordings from wild-type (*w^1118^*), *hist^ko^*, *ninaE>Hdc^RNAi^* (*ninaE-Gal4*/*UAS-Hdc^RNAi^*), *repo>Hdc^RNAi^* (*repo-Gal4*/*UAS-Hdc^RNAi^*), *hist^ko^*;*ninaE>Hdc^RNAi^* (*hist^ko^;ninaE-Gal4*/*UAS-Hdc^RNAi^*), and *hist^ko^*;*repo>Hdc^RNAi^* (*hist^ko^;repo-Gal4*/*UAS-Hdc^RNAi^*) flies at day 1 (top) and day 3 (bottom). Flies were dark-adapted for 1 min and subsequently exposed to a 2-s pulse of orange light. (**B**) Quantitative analysis of the amplitudes of ERG off-transients shown in (A). (**C**) Phototactic behaviors of wild-type, *hist^ko^*, *ninaE>Hdc^RNAi^*, *repo>Hdc^RNAi^*, *hist^ko^*;*ninaE>Hdc^RNAi^*, and *hist^ko^*;*repo>Hdc^RNAi^* flies at day 3. At least 20 flies were used, and five repeats were quantified for each group. Significant differences between mutant and wild-type flies were determined using unpaired *t* tests. (**D**) Histamine was immunolabeled in horizontal sections of heads from wild-type, *hist^ko^*, *ninaE>Hdc^RNAi^*, *repo>Hdc^RNAi^*, *hist^ko^*;*ninaE>Hdc^RNAi^*, and *hist^ko^*;*repo>Hdc^RNAi^* flies at day 3. Scale bar, 50 μm. (**E**) Levels of histamine and carcinine in the heads of wild-type, *hist^ko^*, *ninaE>Hdc^RNAi^*, *repo>Hdc^RNAi^*, *hist^ko^*;*ninaE>Hdc^RNAi^*, and *hist^ko^*;*repo>Hdc^RNAi^* flies at days 1 and 3. Each sample contained 50 heads, and the mean values from three samples were calculated for each genotype. Error bars indicate SEMs, and significant differences between mutant and wild-type flies were determined using unpaired *t* tests.

To ensure rapid signal transmission in the visual system, flies developed two systems for maintaining the histamine pool: (i) the de novo synthesis of histamine, which is mediated by histidine decarboxylase (Hdc), and (ii) histamine reuptake by histamine transporters. We therefore assumed that de novo histamine synthesis could partially compensate for the loss of HisT. To test this hypothesis, we knocked down *Hdc* in the visual system using *Hdc^RNAi^*. Although expressing *Hdc^RNAi^* in compound eyes throughout development via *GMR-Gal4* completely disrupted ERG transients (fig. S6, A and B) (*39*), knocking down *Hdc* in adult photoreceptor cells via the *ninaE-gal4* driver, which turns on ~80 to 90 hours after puparium formation, did not affect visual transmission, despite the fact that *Hdc* mRNA and Hdc protein levels were largely reduced in adult *ninaE>Hdc^RNAi^* flies (Fig. 6, A and B, and fig. S6, C to E). These data suggest that neurotransmitter recycling plays a major role in sustaining synaptic transmission (*39*, *40*). This provides an ideal opportunity to study the contribution of HisT and histamine recycling to synaptic transmission in the absence of histamine de novo synthesis.

We knocked down *Hdc* in adult *hist* mutants using *ninaE>Hdc^RNAi^* and found age-dependent reductions in synaptic transmission (Fig. 6, A and B, and fig. S7C). Reductions in synaptic transmission were light dependent, as off-transients in *hist^KO^,ninaE>Hdc^RNAi^* flies were maintained under dark conditions (fig. S6F). By contrast, knocking down *Hdc* in wild-type flies did not affect ERG response regardless of age (Fig. 6, A and B). As an additional control, we knocked down *Hdc* in glial cells of both wild-type and *hist* mutant flies. Both exhibited normal visual transmission (Fig. 6, A and B). Consistent with these electrophysiological results, *ninaE*-mediated knockdown of *Hdc* in *hist* mutant flies blocked phototactic behaviors, whereas normal phototaxis was observed when *Hdc* was knocked down in adult photoreceptors of wild-type flies, or when Hdc was knocked down in glial cells of *hist* mutant flies (Fig. 6C). We further examined the distribution and concentration of histamine in these flies. Consistent with ERG and phototactic behavior assays, histamine levels were not affected when *Hdc* was knocked down or when *hist* was knocked out. By contrast, knocking down *Hdc* and knocking out *hist* together decreased histamine levels in an age-dependent manner. This indicates that de novo histamine synthesis and histamine recycling work together to maintain histamine pools (Fig. 6, D and E). Although *ninaE*-mediated knockdown of *Hdc* only occurred in R1 to R6 cells, the histamine signals were also reduced in R7 and R8 cells in *hist^ko^,Hdc^RNAi^* flies. We further assessed phototaxis to UV (ultraviolet) light, which reflects the activity of R7 cells. Consistent with the histamine staining results, phototaxis to UV light was unaffected in *hist^ko^* flies (fig. S6G). The reduced levels of histamine in R7 and R8 cells may be due to a failure to maintain an adequate histamine pool in *hist^ko^,Hdc^RNAi^* flies. Alternatively, impairment of synaptic transmission in R1 to R6 cells could affect the function of R7/8 cells (*41*).

Consistent with the role of HisT in histamine recycling, reduced levels of carcinine were also detected in *hist^ko^*;*ninaE>Hdc^RNAi^* flies (Fig. 6E). Together, these results demonstrated that the histamine transporter HisT plays an important role in reusing the neurotransmitter histamine and that this histamine recycling process can be partially compensated for by the de novo synthesis of histamine.

## DISCUSSION

Neurotransmitter clearance is essential for the precise control of neuronal activity and for the maintenance of normal neurotransmitter concentrations. Under normal physiological conditions, the reuptake of most monoamine neurotransmitters (including dopamine, norepinephrine, and serotonin) from the synaptic cleft is mediated by the SLC6 family of transporters, which have high affinity and are ion dependent (*8*). The high specificity and active transport of these uptake-1 transporters ensure low concentrations of extraneuronal neurotransmitters. However, unlike other catecholamines, an ion-dependent and highly specific transmembrane transporter for histamine has not been identified in any system. Histamine is the primary neurotransmitter released by photoreceptors in the fly, and to enable rapid signal transmission, histamine is largely recycled in laminar epithelial glial cells to maintain adequate histamine pools (*25*, *27*, *39*, *42*). Here, we identified transporters enriched in epithelial glia and used an in vitro screen to identify a member of the SLC49 family of transporters, namely, HisT, that is capable of transporting histamine. The histamine transporting activity of HisT is Na^+^ dependent and specific to histamine (i.e., it does not transport carcinine). Thus, HisT is an uptake-1 transporter. By contrast, uptake-2 transporters, such as OCT2, are able to mediate ion-independent transport of both histamine and carcinine at high capacity (*31*). Misexpression of wild-type HisT, but not versions that lacked transporter activity, in photoreceptor cells disrupted visual transmission and phototactic behaviors without affecting the levels of histamine in axons. This suggests that the overexpression of HisT in presynaptic neurons reduces levels of histamine in the synaptic cleft enough to attenuate the activity of postsynaptic neurons. In support of this, carcinine levels were largely decreased, indicating that less histamine entered ebony-expressing glial cells. By contrast, misexpression of wild-type HisT in postsynaptic LMC neurons reduced both histamine and carcinine levels in a transport activity–dependent manner, further indicating that HisT functions in vivo as a histamine transporter.

Known ion-dependent monoamine transporters, such as DAT and NET, are expressed in presynaptic neurons to terminate signal transmission and to enable neurotransmitter reuse by presynaptic neurons. However, these transporters are not found at synapses (*43*, *44*), and therefore, neurotransmitters must diffuse from the synaptic cleft to sites of transport. In the fly visual system, signals must be transmitted at high frequencies and speed, and neurotransmitters must be rapidly cleared from the synaptic cleft. We found that misexpression of HisT in photoreceptors, which are closer to released histamine than epithelial glial cells, disrupted visual transmission, indicating that, under normal conditions, presynaptic neurons do not directly uptake the neurotransmitter histamine. By contrast, OCTs and PMAT transporters are expressed by astrocytes that are near neurons to facilitate the clearance of neurotransmitters (*45*). As these uptake-2 transporters are the only histamine transporters that have been identified, it was proposed that astrocytes are involved in clearing histamine (*10*, *11*). However, the fact that HNMT (histamine *N*-methyltransferase), which inactivates histamine, localizes to neurons suggests that neurons may be involved in clearing histamine (*46*, *47*). In *Drosophila*, Ebony is the enzyme that inactivates histamine, and it is expressed exclusively by epithelial glial cells (*25*, *27*). We show here that the newly identified histamine transporter HisT also localizes to epithelial glial cells, supporting the notion that the clearance of histamine mainly involves glial cells.

**Fig. 7.**
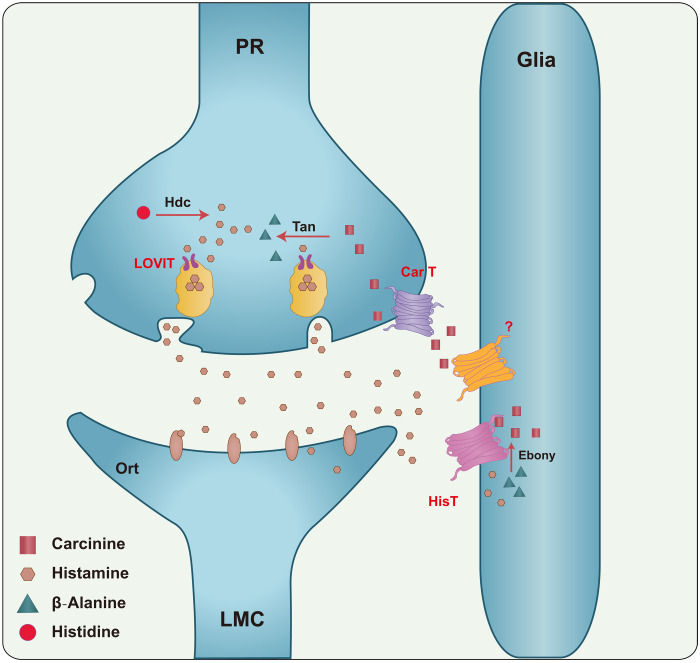
A model of the histamine recycling pathway. Histamine is de novo synthesized by Hdc in photoreceptor cells (PR). Upon light stimulation, PRs release histamine into the synaptic cleft. Released histamine is quickly taken up by the histamine transporter HisT and another unknown histamine transporter into epithelial glial cells, which express Ebony. Histamine is inactivated to carcinine through conjugation to β-alanine catalyzed by Ebony. Carcinine is then released into the synaptic cleft and subsequently taken up via CarT by the PRs, where it is hydrolyzed to regenerate histamine through Tan hydrolase in the PR. Last, histamine is repackaged into synaptic vesicles by LoviT, while β-alanine is transported back to epithelial glia through a BalaT-dependent multicellular trafficking pathway.

Compared to the de novo synthesis pathway, histamine recycling is considered the dominant pathway for maintaining histamine levels in photoreceptors (*39*, *40*). Supporting this, disruption of histamine recycling at any step impairs visual transmission (Fig. 7) (*25*, *27*, *31*–*33*, *48*). Knocking down *Hdc* in the adult eye did not affect photoreceptor synaptic transmission, demonstrating a relatively minor role for de novo synthesis in the synaptic transmission of adult photoreceptors. Similarly, *hist* null mutant flies exhibited normal photoreceptor synaptic transmission, but knocking down *Hdc* in *hist* mutant adult flies disrupted photoreceptor synaptic transmission. In these double mutants, both histamine and carcinine levels were reduced, whereas β-alanine concentration and distribution were unaffected. This is the first demonstration that the de novo synthesis of histamine plays a complementary role in maintaining the histamine pool and sustaining visual transmission. The physiological role of HisT in histamine recycle and maintaining the histamine pool is most important when the synthesis of new histamine is compromised. As a high-affinity transporter, HisT may function to maintain maximal levels of uptake of extracellular histamine by epithelial glia for reuse. Therefore, when histamine levels are low, HisT may play a dominant role in histamine recycling. Supporting this conclusion, when we genetically limited the replenishment of the histamine pool in *ninaE>Hdc^RNAi^* flies, loss of *HisT* gradually reduced histamine levels and disrupted visual transmission. On the basis of these results, we suggest that a previously unidentified, high-affinity uptake-1 histamine transporter, namely, HisT, contributes to histamine recycling through epithelial glial cells.

By identifying the histamine transporter HisT, our study helps to answer an important question: How is histamine transported into epithelial glia? This critical detail will enable the formulation of more complete models of histamine recycling, which is critical for sustaining photoreceptor synaptic transmission (Fig. 7). The fact that HisT is an uptake-1 transporter with high affinity and low capacity suggests that HisT may be able to transport histamine at low concentrations but that the rate of transport may not be sufficient to rapidly clear histamine in this fastest-known signal transduction system. Moreover, knocking out *hist* alone did not affect photoreceptor synaptic transmission and histamine pools, suggesting that there is another histamine transporter that can partially compensate for HisT in histamine recycling. Considering that rapid neurotransmitter clearance is required for high-speed and high-frequency visual transduction, we hypothesize that epithelial glial cells express an uptake-2 transporter that functions to clear histamine from the synapse. This unidentified transporter would play a complementary role with HisT in both histamine clearance and recycling. In mammals, the collaboration between uptake-1 DAT and uptake-2 OCT3 in transporting dopamine supports our hypothesis that uptake-1 and uptake-2 histamine transporters function together in epithelial glial cells (*49*). Similarly, mice lacking *Oct2*, *Oct3*, or *PMAT* do not exhibit large changes in the concentration of histamine or other catecholamines and do not exhibit disruption in histamine-regulated behaviors, such as the sleep-wake cycle and aggression (as seen with the loss of HNMT) (*15*, *19*, *20*, *50*). Furthermore, although uptake-2 transporter inhibitors decrease histamine uptake in rat medial hypothalamic tissues, these inhibitors do not completely abolish histamine transport activity, indicating that an additional uptake-1 transporter is involved in histamine inactivation (*51*). Here, we identify HisT as an uptake-1 transporter with histamine transport activity, finally identifying a substrate-specific histamine transporter. As multiple neurotransmitters are released into photoreceptor cell synapse [e.g., amacrine cells release glutamate (*52*)], an uptake-1 histamine transporter could specifically recognize and transport histamine without affecting other neurotransmitters. As in flies, there may be a specific uptake-1 transporter in the mammalian brain for histamine clearance. Inhibitors of high-selective uptake-1 transporter for norepinephrine, serotonin, and dopamine are frequently used to treat neurological disorders. Thus, the identification of a high-affinity histamine transporter provides a valuable therapeutic target for treating neurological disorders associated with defects in histaminergic neurotransmission (*9*, *53*).

## MATERIALS AND METHODS

### Fly stocks and cultivation

The *Hdc^P217^*, *hs-Cre, M(vas-int.Dm) ZH-2A;M(3xP3-RFP.attP) ZH-86Fb*, and *Df(2R)ED1715* were provided by Bloomington Drosophila Stock Center. The *w^1118^*, *repo-Gal4*, *ninaE-Gal4*, *GMR-Gal4*, and *nos-Cas9* flies were maintained in the laboratory of T.W. at the National Institute of Biological Sciences, Beijing, China. Flies were maintained in 12-hour light/12-hour dark cycles with ~2000 lux illumination at 25°C.

### Generation of *hist* mutant and knock-in flies

The *hist^ko^* mutation was generated using the Cas9/single guide RNA (sgRNA) system (*31*). Briefly, two pairs of guide DNAs targeting the *hist* (CG1358) locus were designed (sgDNA1: CACCTTCGGCTGGGGGGTTA and sgDNA2: AAAACCAACTGCCTGGGAAT) and cloned into the *U6b-sgRNA-short* vector. The plasmids were injected into the embryos of *nos-Cas9* flies, and deletions were identified by PCR using the following primers: 5′-ACACAGAGCTGGTTAGCTAATGGT-3′ (forward) and 5′-CACTTTATCCAAAAACCAACTGCC-3′ (reverse). The *hist^GFP^* knock-in flies were generated as shown in fig. S3. Briefly, a single sgDNA sequence (5′-ATGGACCTGAAGCACAGTGC-3′) was designed and cloned into the *U6b-sgRNA-short* vector. To generate a donor construct, the GFP coding DNA downstream of a 3XP3 promoter was inserted between two fragments from the *hist* genomic sequence (−1023 to −1 and +22 to +982, where +1 represents the translation starting site) and was subcloned into *pDM19* vector. The two plasmids were coinjected into the embryos of *nos-Cas9* flies, and the subsequent GFP-positive progenies were screened. The candidate flies were crossed with *hs-Cre* flies to delete the 3XP3 promoter region, and the *hist^GFP^* knock-in flies were lastly confirmed by PCR of genomic DNA using the following primers: 5′-CCAAATGCAATCGGAATCCGAAAC-3′ (forward) and 5′-GAACTTCAGGGTCAGCTTGCCGTA-3′ (reverse). Both *hist^ko^* and *hist^GFP^* flies were backcrossed to wild-type flies (*w^1118^*) for two generations before performing the experiment.

### Generation of *ebony* and *ort* knock-in flies

The *ebony^mCherry^* and *ebony^Gal4^* knock-in flies were generated as shown in fig. S2. Briefly, an sgDNA sequence (5′-TCAGAATTTGTTATTACCCA-3′) was designed and cloned into the *U6b-sgRNA-short* vector. The *mCherry* or *Gal4* DNA flanking by the *ebony* genomic DNA (−800 to +249 and +311 to +1358, where +1 represents the transcriptional starting site) was subcloned into a donor vector. The two plasmids were coinjected into the embryos of *nos-Cas9* flies. The *ebony^mCherry^* flies were identified by PCR of genomic DNA using the following primers: 5′-CAGCGGCGGATCGGTCGGATATAT-3′ (forward) and 5′-ATGTGCACCTTGAAGCGCATGAAC-3′ (reverse). The *ebony^Gal4^* flies were lastly confirmed by PCR of genomic DNA using the following primers: 5′-CAGCGGCGGATCGGTCGGATATAT-3′ (forward) and 5′-AGAGTAGCGACACTCCCAGTTGTT-3′ (reverse). Both *ebony^mCherry^* and *ebony^Gal4^* flies were backcrossed to wild-type flies (*w^1118^*) for two generations before performing the experiment. The *ort^Gal4^* knock-in flies were generated as shown in fig. S5. Briefly, an sgDNA sequence (5′-TGCATGAAGCACTACGCCAA-3′) was designed and cloned into the *U6b-sgRNA-short* vector. The *Gal4* DNA flanking by two fragments of *ort* genomic DNA (−846 to +125 and +275 to +1292, where +1 represents the transcriptional starting site) was subcloned into a donor vector. The two plasmids were coinjected into the embryos of *nos-Cas9* flies. The *ort^Gal4^* flies were identified by PCR of genomic DNA using the following primers: 5′-ATTGGAGCATCTTGCACTTTGGA-3′ (forward) and 5′-AGAGTAGCGACACTCCCAGTTGTT-3′ (reverse). *ort^Gal4^* flies were backcrossed to wild-type flies (*w^1118^*) for two generations before performing the experiment.

### Generation of plasmid constructs and transgenic flies

Candidate histamine transporters including *hist*, *kcc*, *Nckx30C*, *CG42575*, *Ae2*, *Eaat1*, *CG6356*, and *zyd* complementary DNA (cDNA) sequences were amplified from GH15861, LD02554, GH04818, GH23727, LD27619, FI02126, FI05810, and RE28359 cDNA clones obtained from the Drosophila Genomics Resource Center, Bloomington, IN, USA. The human OCT2 cDNA sequences were synthesized as previously described (*31*). The human FLVCR1 and FLVCR2 cDNA sequences were amplified from I0H56478 and I0H13938 cDNA clones obtained from Invitrogen Ultimate ORF Clones. Their entire coding sequence sequences were subcloned into the pCDNA3 vector (Thermo Fisher Scientific, Carlsbad, USA) for expression in HEK293T cells or the pIB vector (Thermo Fisher Scientific, Carlsbad, USA) for expression in S2 cells. Disease-associated mutations of *hist* construct were generated using primers listed in table S1. To construct *pIB-hist-Myc*, the entire coding sequence of *hist* was subcloned into the *pIB-cMyc* vector between the Acc65 I and Xba I sites. To construct *pIB-hist^L109P^-Myc*, the entire coding sequence of *hist^L109P^* was subcloned into the *pIB-cMyc* vector between the Acc65 I and Xba I sites. To construct *pIB-hist^P252R^-Myc*, the entire coding sequence of *hist^P252R^* was subcloned into the *pIB-cMyc* vector between the Acc65 I and Xba I sites. To construct *pUAS-hist*, the entire coding sequence of *hist* was subcloned into the *pUAST-attB* vector between the Acc65 I and Xba I sites. To construct *pUAS-hist^L109P^-Myc*, *pIB-hist^L109P^-Myc* was cut and subcloned into the *pUAST-attB* vector between the Acc65 I and Xba I sites. To construct *pUAS-hist^P252R^-Myc*, *pIB-hist^P252R^-Myc* was digested and subcloned into the *pUAST-attB* vector between the Acc65 I and Xba I sites. We produced an *Hdc^RNAi^* line as previously described (*54*) by designing a 21-nucleotide short hairpin RNA sequences in 5′ untranslated region (5′UTR) (5′-GCGACAATTGCACAGTATTTA-3′) and cloning them into a *VALIUM20* vector. These constructs were injected into *M(vas-int.Dm) ZH-2A;M(3xP3-RFP.attP) ZH-86Fb* embryos, and transformants were identified on the basis of eye color. The *3xP3-RFP* and *w+* markers were removed by crossing with *P(Cre)* flies.

### RNA extraction and qPCR

For lamina RT-PCR, adult lamina tissues were dissected from 3-day-old flies, and total RNA was extracted using TRIzol reagent (Thermo Fisher Scientific, Carlsbad, USA), followed by TURBO DNA-free deoxyribonuclease treatment (Ambion, Austin, USA). Total cDNA was synthesized using an iScript cDNA synthesis kit (Bio-Rad Laboratories, USA). Three different samples were collected from each genotype. The primer pairs used for RT-PCR were as follows: *rpL32*, 5′-GCCGCTTCAAGGGACAGTATCTG-3′/5′-AAACGCGGTTCTGCATGAG-3′; *hist*, 5′-GCTACAAAGTCTACGCCCGA-3′/5′-GGTGATGCGAAGACCCACT-3′; *ebony*, 5′-ATGCAGTGCAGCTAGAGGAG-3′/5′-GAAACTTAGGTCCCGCTCCA-3′; *ort*, 5′-GCCAAAGGGGAGTTTCAACAA-3′/5′-TCAAGTCATCCGTGGTGTGG-3′; *cart*, 5′-CGCTGTCCATACCCAAGGAA-3′/5′-ATCGCACACTAGATCGAAATCAA-3′; *Hdc*, 5′-GACGGACTCGGGCTATTCAG-3′/5′-TCATCCGCCTCGATGTAACG-3′. Other primer pairs used for RT-PCR are listed in table S1.

### FACS and RNA-seq analysis

Heads of 3-day-old *ebony>GFP* flies and brains of 3-day-old *repo>GFP* flies were collected and dissected. The collected tissues were then digested with papain solution (1 mg/ml; Sigma-Aldrich, P4762) for 1 hour in a thermomixer at 25°C. The samples were mixed every 10 min and centrifuged at 500*g* for 5 min at 4°C after digestion. Papain solution was then removed and washed twice with phosphate-buffered saline at 4°C. The samples were suspended and filtered through a 40-μm membrane into a 5-ml FACS tube. GFP-positive cells were sorted using BD FACSAria II sorter, and RNA was purified using the Arcturus PicoPure RNA isolation kit (Thermo Fisher Scientific, KIT0204) and amplified using an Arcturus RiboAmp HS PLUS RNA amplification kit (Thermo Fisher Scientific, KIT0525). RNA integrity was checked using a 2100 Bioanalyzer (Agilent Technologies, Santa Clara, CA). The mRNAs were enriched using oligo magnetic beads (Thermo Fisher Scientific, USA) and fragmented to ∼150- to 250-bp products. The cDNAs were synthesized using random hexamer primers and purified using a MinElute PCR purification kit (QIAGEN, Valencia, CA). The 42-cycle single-end sequencing was performed using an Illumina Genome Analyzer IIx, and CASAVA pipeline v1.8 was then used for sequence extraction and filtering. RNA-seq reads were mapped to the fly genome using the Tophat (v2.0.8b) software and the Ensembl genome annotation dataset (*Drosophila*_melanogaster.BDGP5.71.gtf). Expression of genes in the RNA-seq data was measured by per kilobase of exon per million fragments mapped using Cufflinks (v2.1.1) software.

### Histamine uptake assay

Histamine uptake was measured as described using [^3^H]-histamine [ring, methylenes-^3^H(N)] dihydrochloride (10 to 40 Ci/mM; American Radiolabeled Chemicals, Saint Louis, USA) (*48*). Briefly, HEK293T cells were cultured in 12-well plates and transfected with candidate transporters including HisT using vigofect reagent (Vigorous Biotechnology, Beijing, China). The transfected cells were washed with 1 ml of extracellular fluid (ECF) buffer consisting of 120 mM NaCl, 25 mM NaHCO_3_, 3 mM KCl, 1.4 mM CaCl_2_, 1.2 mM MgSO_4_, 0.4 mM K_2_HPO_4_, 10 mM d-glucose, and 10 mM Hepes (pH 7.4) at 37°C. For Na^+^-free ECF buffer, NaCl was constituted with 120 mM choline chloride. For Cl^−^-free ECF buffer, NaCl was constituted with 120 mM sodium gluconate. Uptake assays were initiated by applying 200 μl of ECF buffer containing 7400 becquerel (Bq) [^3^H]-histamine (2.5 μM) at 37°C. After 30 min, the reaction was terminated by removing the solution, and cells were washed with 1 ml of ice-cold ECF buffer. The cells were then solubilized in 1 N NaOH and subsequently neutralized. An aliquot was taken to measure radioactivity and protein content using a liquid scintillation counter and a DC protein assay kit (Bio-Rad, USA), respectively. To perform histamine competition assays, 7400 Bq [^3^H]-histamine in combination with specific monoamines at indicated concentration was added into ECF buffer. The mixture was incubated for 30 min, and the continuous procedures are similar to the histamine transport assay.

### Immunohistochemistry

Cells cotransfected with HisT-Myc and BalaT-mCherry were incubated with rabbit anti-Myc antibody (1:200; Sigma-Aldrich) and rat anti-mCherry antibody (1:200; Chromotek, Germany). Goat anti-rabbit immunoglobulin G (IgG) conjugated to Alexa 488 (1:500; Thermo Fisher Scientific, USA; RRID:AB_143165) and goat anti-rat IgG conjugated to Alexa 568 (1:500; Invitrogen, USA; RRID:AB_2534121) were used as secondary antibodies. Head sections (10 μm thick) were prepared from adult flies that were frozen in O.C.T. medium (Tissue-Tek, Torrance, USA). Immunolabeling was performed on cryosections with rat anti-LOVIT (1:100) (*55*), rat anti-mCherry antibody (1:200; Chromotek, Germany), or rabbit anti-GFP (1:200; Thermo Fisher Scientific, USA) as primary antibodies. For histamine immunolabeling, sections were fixed with 4% 1-ethyl-3-(3-dimethylaminopropyl) carbodiimide (Thermo Fisher Scientific, USA), and the rabbit anti-histamine (1:100; ImmunoStar, USA) antibody was preadsorbed with carcinine, as previously reported (*31*). For carcinine immunolabeling, fly heads were fixed with 4% paraformaldehyde and immersed in 12% glucose overnight at 4°C. Fly head sections (10 μm thick) were prepared from adults that were frozen in O.C.T. medium (Tissue-Tek, Torrance, USA). The rabbit anti-histamine (1:100; ImmunoStar, USA) antibody was preadsorbed with histamine. Goat anti-rabbit IgG conjugated to Alexa 488 (1:500; Thermo Fisher Scientific, USA), goat anti-mouse IgG conjugated to Alexa 488 (1:500; Thermo Fisher Scientific, USA), goat anti-rabbit IgG conjugated to Alexa 568 (1:500; Thermo Fisher Scientific, USA), and goat anti-rat IgG conjugated to Alexa 647 (1:500; Thermo Fisher Scientific, USA) were used as secondary antibodies. The images were recorded with a Nikon A1-R confocal microscope.

### Western blotting

Fly heads were homogenized in SDS sample buffer with a pellet pestle (Kimble Chase, Vineland, NJ, USA), and the proteins were fractionated by SDS–polyacrylamide gel electrophoresis. Proteins from the gels were then transferred onto Immobilon-FL transfer membranes (Millipore, Danvers, MA, USA) in tris-glycine buffer. The rat anti-Hdc (1:1000) and mouse anti-actin (1:2000; Santa Cruz Biotechnology) were used as primary antibodies. The blots were subsequently probed with IRDye 800 IgG and IRDye 680 IgG (LI-COR Biosciences). Signals were detected with an Odyssey infrared imaging system (LI-COR Biosciences).

### ERG recordings

ERGs were recorded as previously described (*31*). Briefly, two glass microelectrodes were filled with Ringer’s solution, and placed on the surfaces of the compound eye and thorax (one each surface). The light intensity was ~0.3 mW/cm, and the wavelength was ~550 nm (source light was filtered using an FSR-OG550 filter). The electrical signals were amplified with a Warner electrometer IE-210 and were recorded with a MacLab/4 s A/D converter and Clampex 10.2 program (Warner Instruments, Hamden, USA). All recordings were carried out at 25°C.

### The phototaxis assay

Flies were dark-adapted for 15 min before the phototaxis assay, as previously described (*31*). A transparent glass tube 20 cm in length and 2.5 cm in diameter was used. A white light source (with a light intensity of ~6000 lux) or a UV light source (365 nm, 2000 lux) was placed at one end of the glass tube. The tube was placed horizontally in the dark, and dark-adapted flies were gently tapped into the other end of the tube (away from the light source). The light was turned on, and the number of flies that walked past an 11-cm mark on the tube within 30 s was counted. The phototaxis index was calculated by dividing the number of flies that walked past the mark by the total number of flies. Five groups of flies were collected for each genotype, and three repeats were made for each group. Each group consisted of at least 20 flies. Results were expressed as the mean for the five groups.

### Liquid chromatography–mass spectrometry

Liquid chromatography–mass spectrometry was performed as previously reported (*48*). Briefly, the Dionex Ultimate 3000 UPLC system was coupled to a TSQ Quantiva Ultra triple-quadrupole mass spectrometer (Thermo Fisher Scientific, CA) and equipped with a heated electrospray ionization probe in negative ion mode. Extracts were separated by a Fusion-RP C18 column (2 × 100 mm, 2.5 μm, Phenomenex). Data were acquired in selected reaction monitoring for histamine, carcinine, and β-alanine with transitions of 112/95.2, 183/95, and 90/72, respectively. Both precursor and fragment ions were collected with a resolution of 0.7 full width at half maximum. The source parameters (spray voltage, 3000 V; ion transfer tube temperature, 350°C; vaporizer temperature, 300°C; sheath gas flow rate, 40 Arb; auxiliary gas flow rate, 20 Arb; collision induced dissociation gas, 2.0 mtorr) are set. Data analysis and quantification were performed using the Xcalibur 3.0.63 software (Thermo Fisher Scientific, CA). Each sample contained 50 *Drosophila* heads. Three samples were measured for each genotype, and the mean values from three samples were calculated.

### Statistical analysis

All experiments were repeated as indicated in each figure legend. All statistical analyses were performed using GraphPad Prism 8. The variations of data were evaluated as means ± SEM. The statistical significance of the differences between two groups was measured by the unpaired two-tailed Student’s *t* test and one-way analysis of variance (ANOVA). A value of *P* < 0.05 was considered statistically significant. *P* value, SEM, and number are as indicated in each figure and legend.

## Supplementary Materials

### Other Supplementary Material for this manuscript includes the following:

Datasets S1 to S3

## Notes

View/request a protocol for this paper from *Bio-protocol*.
