# Supplementary material for: Drosophila HisT is a specific histamine transporter that contributes to histamine recycling in glia
Source: Sci Adv. 2022 Oct 26;8(43):eabq1780. doi: 10.1126/sciadv.abq1780 (PMC9604546; doi:10.1126/sciadv.abq1780)
Supplement: Supplementary file 1 — Figs. S1 to S7 [file sciadv.abq1780_sm.pdf]

Science Advances
AAAS

Supplementary Materials for

Drosophila HisT is a specific histamine transporter that contributes to histamine recycling in glia

Jun Xie *et al.*

Corresponding author: Tao Wang, wangtao1006@nibs.ac.cn

Sci. Adv. **8**, eabq1780 (2022)
DOI: 10.1126/sciadv.abq1780

The PDF file includes:

Figs. S1 to S7
Legends for datasets S1 to S3

Other Supplementary Material for this manuscript includes the following:

Datasets S1 to S3

Fig S1

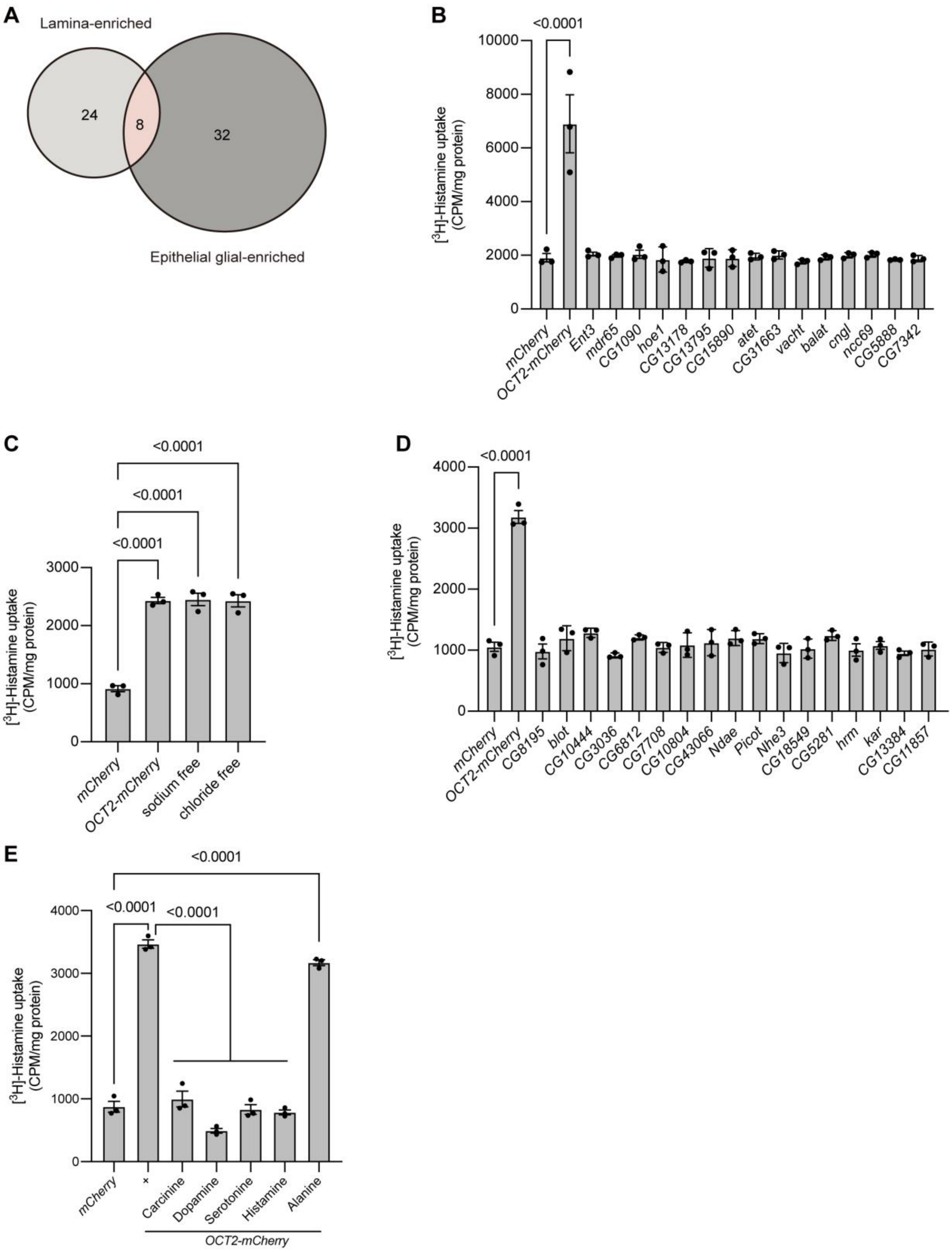
A
Lamina-enriched
24
8
32
Epithelial glial-enriched
B
<0.0001
[3H]-Histamine uptake (CPM/mg protein)
10000
8000
6000
4000
2000
0
mCherry
OCT2-mCherry
Ent3
mdr65
CG1090
hoe1
CG13178
CG13795
CG15890
atet
CG31663
vacht
balat
cngl
ncc69
CG5888
CG7342
C
<0.0001
<0.0001
<0.0001
[3H]-Histamine uptake (CPM/mg protein)
3000
2000
1000
0
mCherry
OCT2-mCherry
sodium free
chloride free
D
<0.0001
[3H]-Histamine uptake (CPM/mg protein)
4000
3000
2000
1000
0
mCherry
OCT2-mCherry
CG8195
blot
CG10444
CG3036
CG6812
CG7708
CG10804
CG43066
Ndae
Picot
Nhe3
CG18549
CG5281
hrm
kar
CG13384
CG11857
E
<0.0001
<0.0001
<0.0001
[3H]-Histamine uptake (CPM/mg protein)
4000
3000
2000
1000
0
mCherry
+
Carcinine
Dopamine
Serotonine
Histamine
Alanine
OCT2-mCherry

Fig. S1.

Screening for histamine transporters. (A) Venn diagram exhibits numbers of lamina-enriched and epithelial-glial-enriched transporter genes, identified by RT-PCR (Figure 1A) and RNA-seq (Figure 1B), respectively. Eight putative transporter genes including *CG1358*, *Kcc*, *Nckx30C*, *CG42575*, *Ae2*, *Eaat1*, *CG6356* and *Ent3* are identified by both methods. (B-C) HEK293T cells transiently expressing one of lamina-enriched transporters (B) or epithelial-enriched transporters (C) were exposed to [^{3}H]-histamine, which was added to the ECF buffer. Human OCT2 and mCherry (red fluorescent protein) were used as positive and negative control, respectively. The results given are the mean values ± SEM of three experiments. (D) OCT2 transporting histamine independent of Na^+ and Cl^-. HEK293T cells transiently expressing OCT2-mCherry were exposed to [^{3}H]-histamine, incubated with ECF, Na^+-free ECF, and Cl^--free ECF buffer, respectively. mCherry was used as control. The results given are the mean values ± SEM of three experiments. (E) OCT2 is a non-specific histamine transporter. HEK293T cells transiently expressing competition assays using [^{3}H]-histamine in combination with different monoamines including histamine, carcinine, dopamine and serotonin at higher concentration (0.1 mM for serotonin and dopamine, 0.5 mM for histamine and carcinine vs. 2.5 µM [^{3}H]-histamine), [^{3}H]-histamine in combination with 0.5 mM alanine was used as control. The results given are the mean values ± SEM of three experiments.

Fig S2

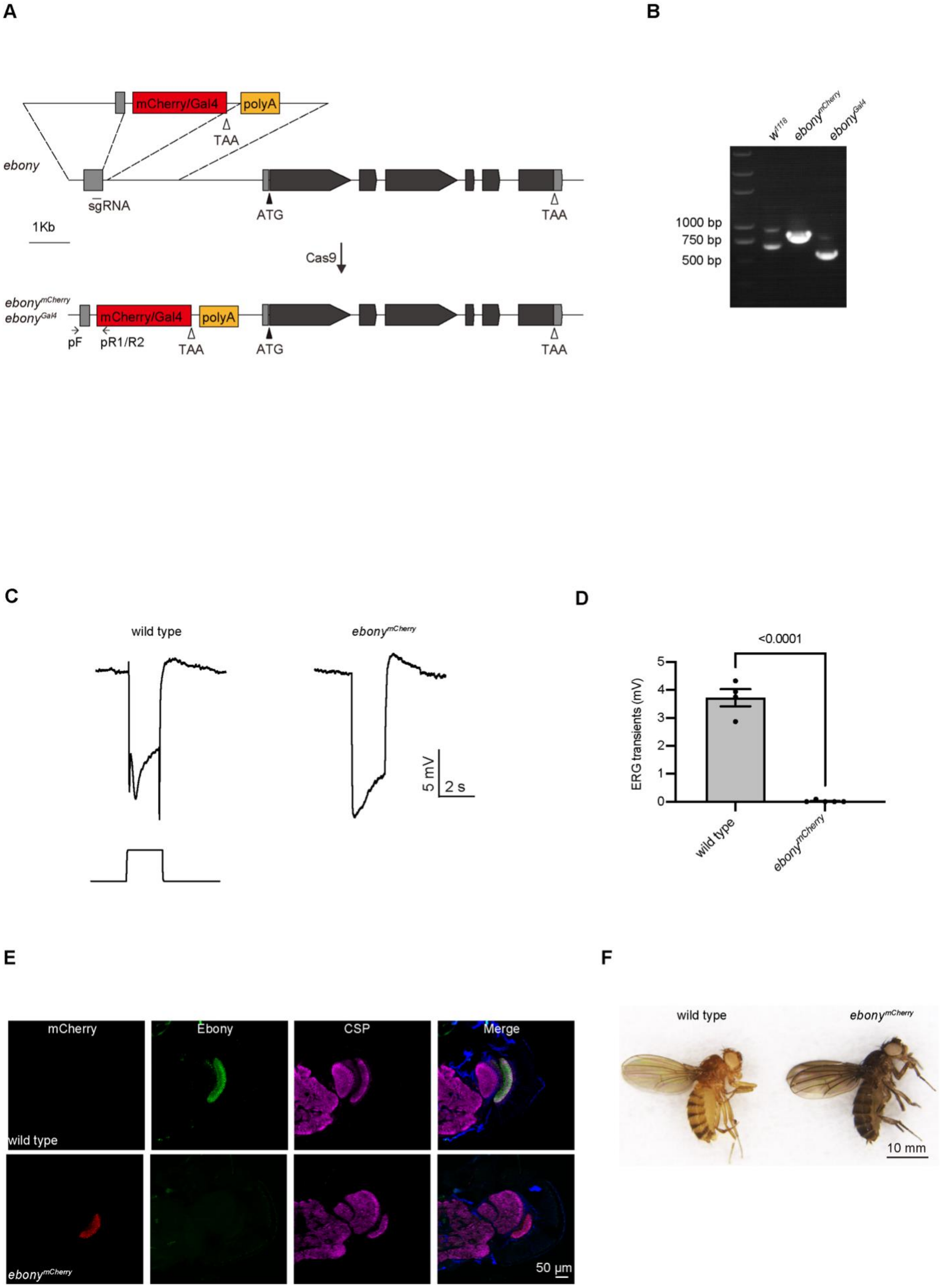
A
mCherry/Gal4
polyA
TAA
ebony
sgRNA
ATG
TAA
1Kb
Cas9
$ebony^{mCherry}$
$ebony^{Gal4}$
mCherry/Gal4
polyA
pF
pR1/R2
TAA
ATG
TAA
B
w^{1118}
$ebony^{mCherry}$
$ebony^{Gal4}$
1000 bp
750 bp
500 bp
C
wild type
$ebony^{mCherry}$
5 mV
2 s
D
<0.0001
ERG transients (mV)
5
4
3
2
1
0
wild type
$ebony^{mCherry}$
E
mCherry
Ebony
CSP
Merge
wild type
$ebony^{mCherry}$
50 µm
F
wild type
$ebony^{mCherry}$
10 mm

Fig. S2.

Generation of *ebony*^{mCherry} and *ebony*^{Gal4} flies. (A) Schematics for generating *ebony*^{mCherry} and *ebony*^{Gal4} flies. The mCherry fluorescent protein or Gal4 transcriptional factor was inserted into the *ebony* genomic locus using CRISPR/Cas9-mediated homologous recombination. The knock-in PCR primers (arrows, pF and pR1/R2) were used to identify the *ebony*^{mCherry} or *ebony*^{Gal4} knock-in flies. (B) Genomic PCR products from wild-type (w^{1118}), *ebony*^{mCherry} and *ebony*^{Gal4} knock-in flies show successful gene targeting. (C) ERG recordings from wild-type (w^{1118}), and *ebony*^{mCherry} flies. Young flies (<3 days after eclosion) were dark adapted for 1 min and subsequently exposed to a 2 s pulse of orange light. (D) Quantitative analysis of the amplitudes of ERG off-transient shown in (C). (E) Cryosections of wild-type (w^{1118}) and *ebony*^{mCherry} flies were labeled for mCherry (Red), Ebony (Green), and CSP (Magenta). Scale bar, 50 µm. (F) The body color of *ebony*^{mCherry} flies were darker than wild-type controls. Scale bar, 10 mm.

Fig S3

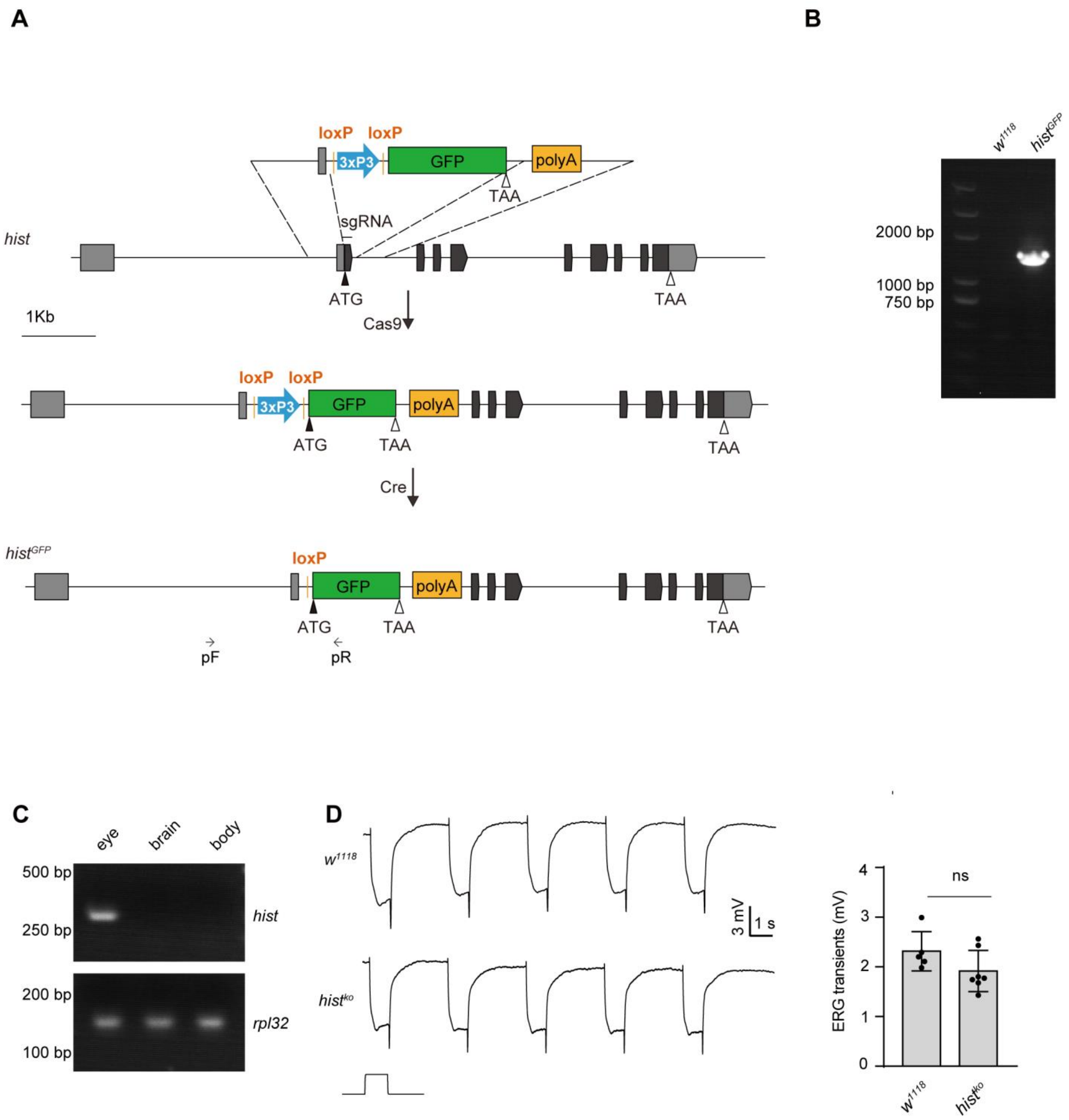

Fig. S3.

Generation of *hist*^{GFP} flies. (A) Schematics for generating *hist*^{GFP} flies. The green fluorescent protein GFP, driven by an eye-specific 3XP3 promoter, was inserted into the *hist* genomic locus using CRISPR/Cas9-mediated homologous recombination. The GFP-positive progenies were screened and the 3XP3 promoter region was subsequently removed by Cre recombinase. PCR primers (arrows, pF and pR) were used to confirm the final *hist*^{GFP} knock-in flies. (B) Genomic PCR products from wild-type (*w*^{1118}) and *hist*^{GFP} knock-in flies show successful gene targeting. (C) HisT was expressed specifically in the fly eye. Fly eye including retina and lamina, brain and

body were dissected, total RNAs were extracted, and RT-PCR experiments were performed. (D) ERG recordings from wild-type (*w*^{1118}) and *hist*^{ko} flies. Young flies (<3 days after eclosion) were dark adapted for 1 min and subsequently exposed to 5 pulses of orange light (1 s pulses with 3 s intervals). Off-transients of the last ERG response were quantified.

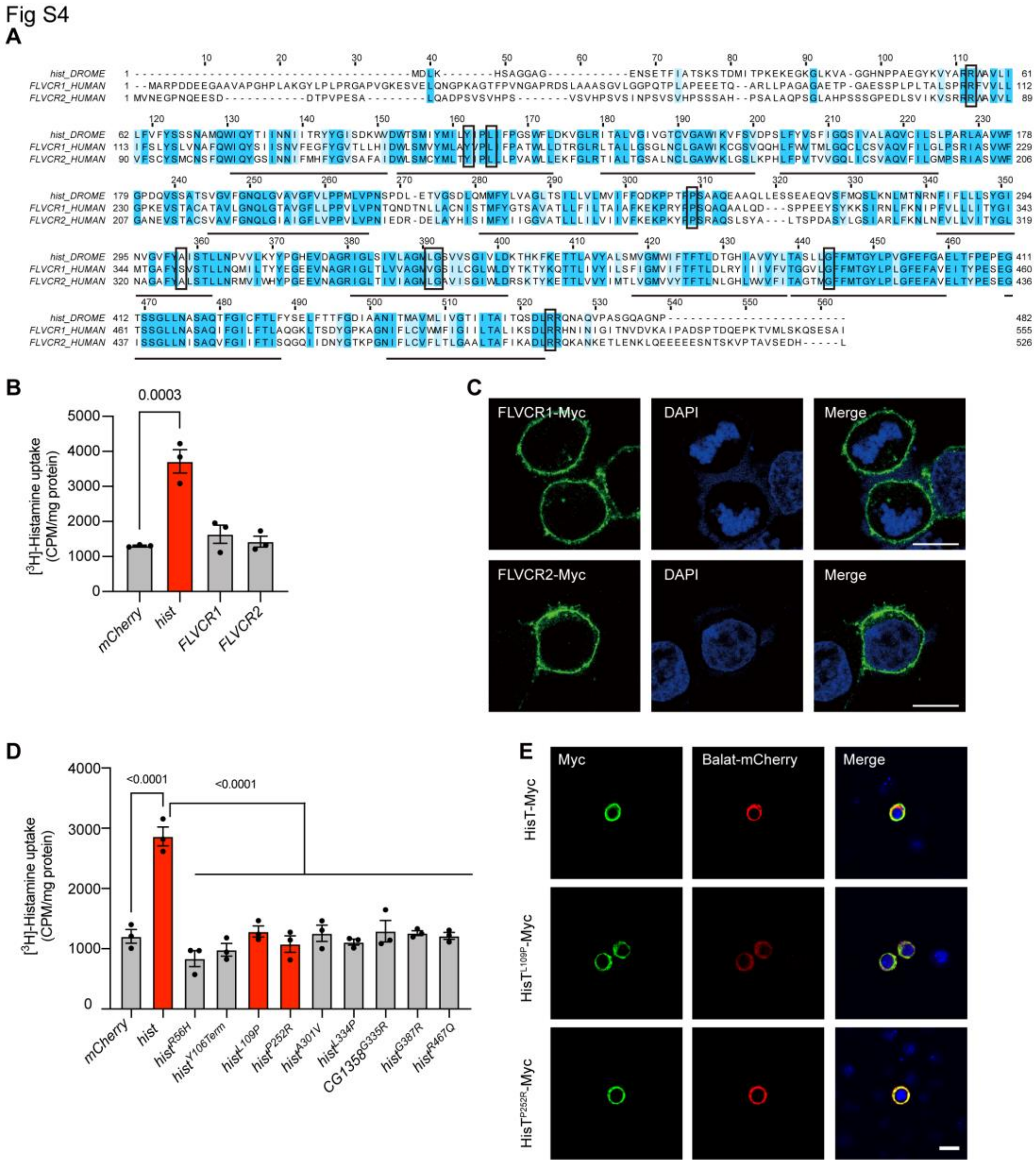

Fig. S4.

Mutation of conserved HisT residues affects histamine uptake activity. (A) Alignment of the *Drosophila* HisT amino acid sequence with human FLVCR1 and human FLVCR2. Identical residues, found in at least two proteins, are indicated in blue color. HisT is 51.02% identical to human FLVCR1 and 52.37% identical to human FLVCR2. Boxes indicates the conserved disease-related mutation sites found in FLVCR1 and FLVCR2. Transmembrane domains are underlined. (B-D) HEK293T cells transiently expressing (B) FLVCR1 and FLVCR2 or (D) mutated HisT were exposed to [^{3}H]-histamine. mCherry and wild-type HisT were used as negative and positive controls, respectively. The results given are the mean values ± SEM of three experiments. (C) FLVCR1 and FLVCR2 localized to the plasma membrane. HEK293T cells transiently expressing Myc-tagged FLVCR1 and FLVCR2 were labeled using antibodies against Myc (green) and DAPI (blue). Scale bar, 10 μm. (E) Both wild-type and mutant HisTs were localized to the plasma membrane. S2 cells were transiently co-transfected with mCherry-

tagged BalaT and Myc-tagged HisT/$HisT^{L109P}$/$HisT^{P252R}$, and then labeled with Myc antibody (green), mCherry antibody (red), and DAPI (blue). Scale bar, 10 μm.

Fig S5

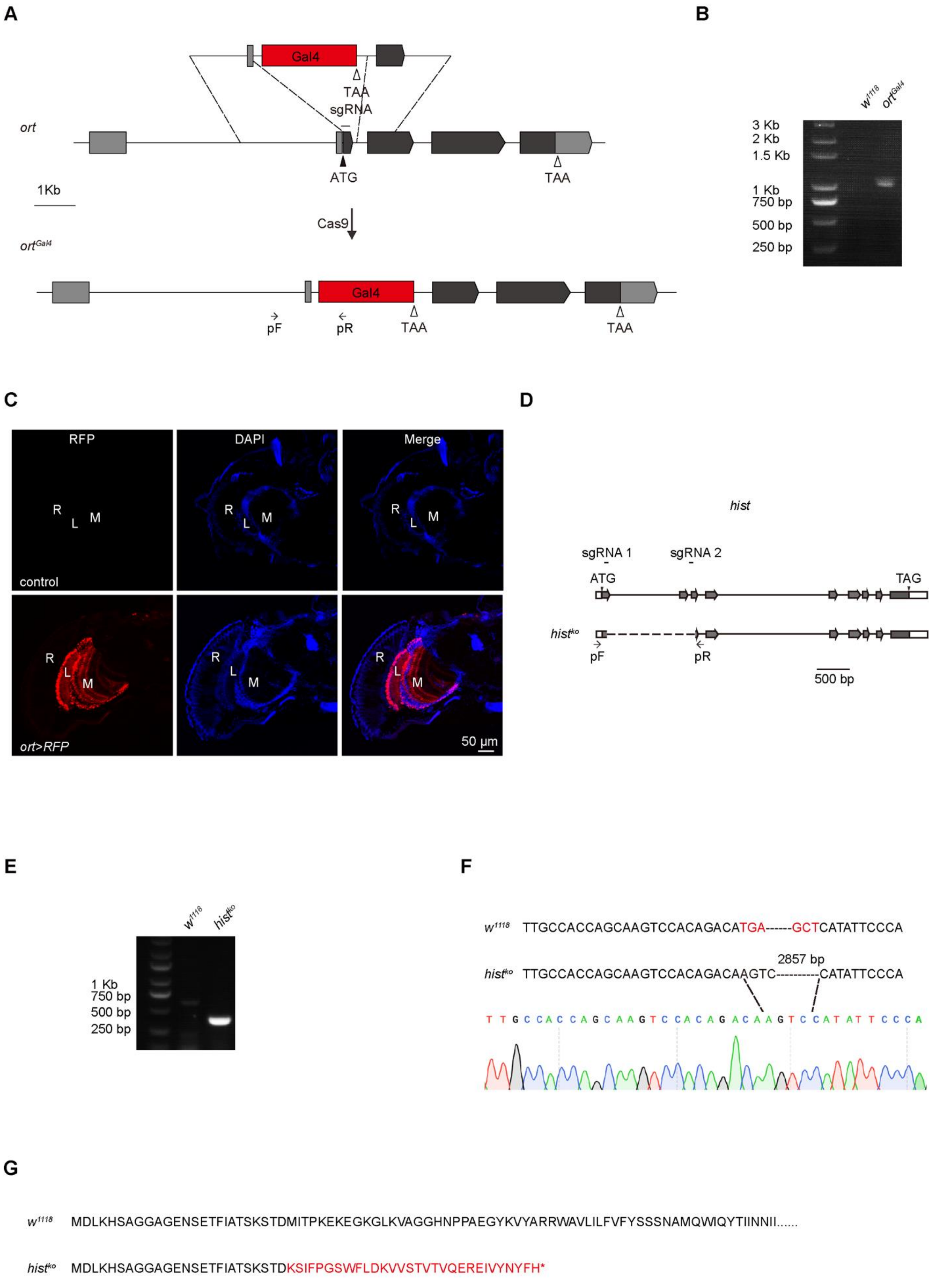
A
Gal4
TAA
sgRNA
ort
ATG
TAA
1Kb
Cas9
ort^Gal4
Gal4
pF
pR
TAA
TAA
B
w1118
ortGal4
3 Kb
2 Kb
1.5 Kb
1 Kb
750 bp
500 bp
250 bp
C
RFP
DAPI
Merge
R
L
M
control
ort>RFP
50 µm
D
hist
sgRNA 1
sgRNA 2
ATG
TAG
histko
pF
pR
500 bp
E
w1118
histko
1 Kb
750 bp
500 bp
250 bp
F
w1118 TTGCCACCAGCAAGTCCACAGACATGA------GCTCATATTCCCA
2857 bp
histko TTGCCACCAGCAAGTCCACAGACAAGTC----------CATATTCCCA
TTGCCACCAGCAAGTCCACAGACAAGTCCATATTCCCA
G
w1118 MDLKHSAGGAGENSETFIATSKSTDMITPKEKEGKGLKVAGGHNPPAEGYKVYARRWAVLILFVFYSSSNAMQWIQYTIINNII......
histko MDLKHSAGGAGENSETFIATSKSTDKSIFPGSWFLDKVVSTVTVQEREIVYNYFH*

Fig. S5.

Generation of *ort*^{Gal4} and *hist*^{ko} flies. (A) Schematics for generating *ort*^{Gal4} flies. The *Gal4* sequence was inserted into the *ort* genomic locus using CRISPR/Cas9-mediated homologous recombination. PCR primers (arrows, pF and pR) were used to identify the *ort*^{Gal4} knock-in flies. (B) Genomic PCR products from wild-type (*w*^{1118}) and *ort*^{Gal4} knock-in flies show successful gene targeting. (C) Cryosections of *ort*^{Gal4}*>RFP* and control (*UAS-RFP*) flies labeled with RFP (red) and DAPI (blue) verify the expression pattern of the *ort*^{Gal4}. (D) Schematic for *hist* knock-out by sgRNA targeting. The organization of the *hist* locus and the expected structures of *hist*^{ko} alleles are shown. Boxes represent exons with the coding region between ATG and TAG. Arrows indicate the primers used for genomic PCR. (E) PCR products obtained from *hist*^{ko} mutant show successful gene deletions. (F) Verification of the *hist*^{ko} locus by DNA sequencing. The *hist*^{ko} deletion mutation eliminated 2857 bp. This leads to a frame shift of the spliced *hist*^{ko} mRNA at the truncated site as shown in (G). (G) Estimated translated protein sequences of wild-type (*w*^{1118}) and *hist*^{ko} flies from the *hist* locus.

Fig S6

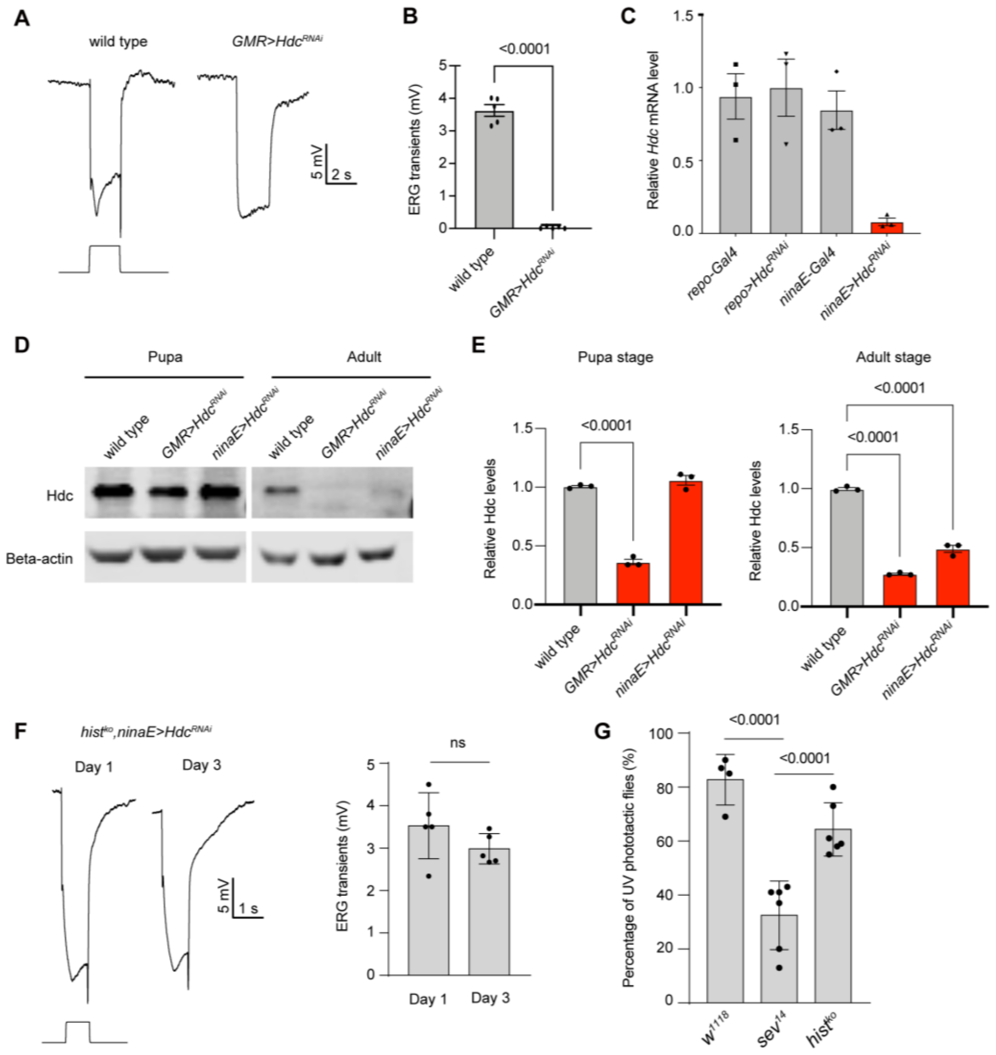

Fig. S6.

Verification of *Hdc*^{RNAi} flies. (A) ERG recordings from wild-type (*w*^{1118}), and *GMR>Hdc*^{RNAi} flies. Young flies (<3 days after eclosion) were dark adapted for 1 min and subsequently exposed to a 2 s pulse of orange light. (B) Quantitative analysis of the amplitudes of ERG off-transients shown in (A). (C) Relative *Hdc* mRNA levels of adult flies. One-day-old *repo-Gal4*, *repo>Hdc*^{RNAi} (*repo-Gal4*/*UAS-Hdc*^{RNAi}), *ninaE-Gal4* and *ninaE>Hdc*^{RNAi} (*ninaE-Gal4*/*UAS-Hdc*^{RNAi}) flies were used for RNA extraction. (D) Western blot of Hdc in wild-type (*UAS-Hdc*^{RNAi}), *GMR>Hdc*^{RNAi}, and *ninaE>Hdc*^{RNAi} flies at pupa stages and adult stages. Pupa eyes and adult heads were dissected at ~70 hr APF and 1 day after eclosion, respectively. (E) Quantification of relative Hdc levels from 3 biological replicates. Levels of Hdc protein were normalized to beta-actin. (F) ERG recordings and quantitative analysis of the amplitude of ERG off-transients from dark raised *hist*^{ko},*ninaE>Hdc*^{RNAi} flies. Flies were maintained in constant dark for 3 days and then exposed to a 1 s pulse of orange light. Amplitude of the ERG off-transients were quantified and compared with those of 1-day-old flies. (G) UV phototactic behavior of wild-type (*w*^{1118}), *sev*^{14}, and *hist*^{ko} flies. *sev*^{14} mutants were used as a positive control. At least 20

3-day-old flies were used, and five trials were quantified for each group. Significant differences between mutant and wild-type flies were determined using the unpaired t-test.

Fig S7

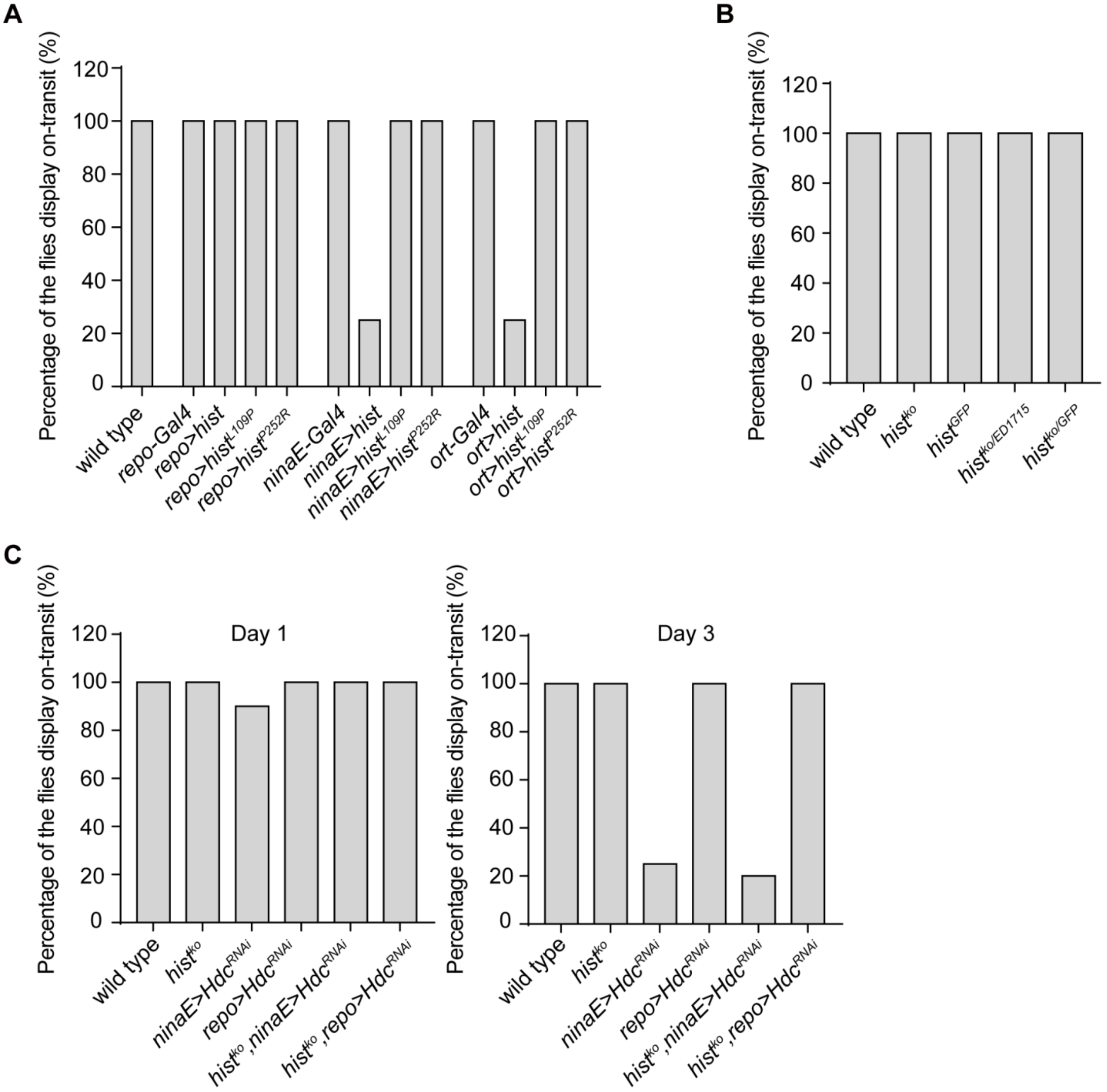

Fig. S7.

Quantification of ERG ON transients. The percentage of flies (genotypes indicated) with ON transients were quantified. ERG profiles from at least 8 flies of each genotype were scored. (A) The percentage of flies corresponding to those in Figure 3. (B) The percentage of flies corresponding to those in Figure 5. (C) The percentage of flies corresponding to those in Figure 6.

Supplementary dataset 1 (abq1780_Suppl. Excel_seq1_v1.xlsx): **Sequence of primer sets used in the study**.
Supplementary dataset 2 (abq1780_Suppl. Excel_seq2_v1.xlsx) : **Primary data used in Statistics**.
Supplementary dataset 3 (abq1780_Suppl. Excel_seq3_v1.xlsx): **RNA-Seq data from epithelial glial cells**. RNA-seq was performed on FACS-isolated GFP-positive epithelial glial cells of *ebony>GFP* flies and glial cells from central brain region of *repo>GFP* flies.
